# Pseudoscientific beliefs and psychopathological risks increase after COVID-19 social quarantine

**DOI:** 10.1186/s12992-020-00603-1

**Published:** 2020-07-30

**Authors:** Álex Escolà-Gascón, Francesc-Xavier Marín, Jordi Rusiñol, Josep Gallifa

**Affiliations:** Faculty of Psychology, Education and Sport Sciences (FPCEE Blanquerna), Ramon Llull University, 34 Císter St., 08022 Barcelona, Spain

**Keywords:** COVID-19, SARS-CoV-2, Psychotic phenotype, Pseudoscientific beliefs, Psychotic disorders

## Abstract

**Background:**

The health crisis caused by COVID-19 has led many countries to opt for *social quarantine* of the population. During this quarantine, communication systems have been characterized by *disintermediation*, the *acceleration of digitization* and an *infodemic* (excess and saturation of information). The following debate arises: Do the levels related to *the psychotic phenotype* and *pseudoscientific beliefs* related to the interpretation of information vary before and after social quarantine?

**Objectives:**

This research aims to examine the psychological effects of social quarantine on the psychotic phenotype and pseudoscientific beliefs-experiences of the general nonclinical population. The following hypothesis was posed: social quarantine alters the levels of magical thinking, pseudoscientific beliefs and anomalous perceptions due to quarantine.

**Methods:**

A *pre- and posttest* analysis design was applied based on the difference in means, and complementary *Bayesian* estimation was performed. A total of 174 Spanish subjects responded to different questionnaires that evaluated psychopathological risks based on psychotic phenotypes, pseudoscientific beliefs and experiences before and after quarantine.

**Results:**

Significant differences were obtained for the variables *positive psychotic symptoms, depressive symptoms,* and certain *perceptual alterations* (e.g., *cenesthetic perceptions*), and a significant increase in pseudoscientific beliefs was also observed. The perceptual disturbances that increased the most after quarantine were those related to *derealization* and *depersonalization*. However, *paranoid perceptions* showed the highest increase, doubling the initial standard deviation. These high increases could be related to the delimitation of physical space during social quarantine and distrust towards information communicated by the government to the population. Is it possible that social alarmism generated by the excess of information and pseudoscientific information has increased paranoid perceptual alterations?

**Conclusions:**

Measures taken after quarantine indicate that perceptual disturbances, subclinical psychotic symptoms and beliefs in the pseudoscience have increased. We discuss which elements of quarantine coincide with the social marginality theory and its clinical repercussions.

## Introduction

Tolerance to uncertainty regarding the future is conditioned and moderated by the degree of control that the subject perceives over what happens in the environment [1]. One of the psychological mechanisms that is activated with the aim of seeking and increasing the feeling of control is magical thinking [2]. Among the most frequent expressions of magical thinking are beliefs that contradict the laws and bases of the current scientific knowledge. These beliefs are usually called pseudoscientific beliefs [3]. In this case, the *Scientific Unexplained Beliefs Model* (hereafter SUB) justifies the social and psychological functionality of pseudoscientific beliefs as a way to feel safe and find an explanation or meaning to the uncertain circumstances that occur throughout life [4]. However, pseudoscientific beliefs - as in most belief systems - also allow the subject to make decisions and take actions that generate behavioral responses whose consequences can affect the mental health of people [5].

### Social, health and theoretical background

In recent months, many countries have been severely affected by the pandemic caused by the SARS-CoV-2 virus [6]. One of the most frequent legislative measures was the social quarantine of the population in their homes and the cessation of economic activities considered nonessential [7, 8]. In this line, the media reported that during the first week of quarantine, some products related to personal hygiene were exhausted in the respective establishments [9]. Although *sanitary masks* and *hydroalcoholic gels* were the first products to disappear, it was reported that toilet paper had also run out [10, 11]. This is the case in most European countries but also includes the United States and Australia, in which some people also bought this product en masse [12]. This type of behavior is classified into *compulsive behaviors* related to fear, anxiety and magical thinking [13, 14]. Similarly, some studies also suggest that they are responses to the need to seek control [15]. Other studies indicate that this extraordinary social situation produced by COVID-19 has generated an increase in magical beliefs and *herd behavior*, which is correlated with the increase in perceived stress during quarantine [16, 17].

The consequences of pseudoscientific beliefs on the health of people were analyzed and investigated from multiple perspectives [4, 18]. These perspectives can be summarized in two models: the first model is based on the psychopathological and symptomatic effects that pseudoscientific beliefs produce in patients [19–23]. Most studies conclude that pseudoscientific beliefs represent an attribute of *the psychotic phenotype*, which is included within the *psychosis continuum* model [24, 25]. At the statistical and epidemiological level, its effects can be synthesized in two points: on the one hand, in an increased probabilistic risk of contracting or developing a future psychotic picture (e.g., paranoid pictures) [26] and, on the other hand, in the clinical or subclinical development of the *attenuated psychotic symptoms syndrome* [27, 28]. This is a relatively new classification included in the DSM-5 that is being studied [29]. In any case, according to this perspective, pseudoscientific beliefs would not represent adaptive models of thought or systems of meanings for the patient and, therefore, would constitute behaviors preferably to be extinguished during the therapeutic course of treatment [30]. It is important to note that the medical conception of mental health has been widely criticized by some research [31]. The problem of the psychopathological perspective is that clinical judgment is often confused with *moral judgment* on the patient’s own beliefs, which determines what is “correct” (functional) versus “incorrect” (dysfunctional) [4, 32]. The mixture of moral and clinical judgment incurs in the *Naturalistic Fallacy* [33, 34]: *- Pseudoscientific beliefs are dysfunctional* (imperative argument); then, − *it is not correct that a person or patient can have pseudoscientific beliefs because they are dysfunctional* (fallacious argument). The separation between decisions involving clinical judgment and moral assessments is essential if the respect and freedom of the patient is to be guaranteed [31].

The second model is outside the psychopathological framework, but within this conception, pseudoscientific beliefs are also understood as cognitive errors or perception biases [5, 35]. This perspective includes perceptual distortion and cognitive styles [36]. In fact, some studies concluded that subjects that believe in *pseudosciences* develop causal illusions more frequently and more heightened than nonbelieving subjects [37]. The psychobiological function of perceptual distortion is based on survival: if the cause of a phenomenon is known, the cause itself and the respective phenomenon could be prevented; this would allow anticipating environmental threats and finding answers that would guarantee the survival of the species [5, 37, 38]. In this area, the most studied perceptual distortions are causal illusions and *pareidolia* [39], which is also very common in believers in pseudoscience [40].

The *social marginality theory* explains the production of pseudoscientific beliefs as a consequence of the personal and geographic isolation of some communities [41]. According to some studies, the greater the social isolation, the higher the levels of magical thinking that individuals in the respective communities who would remain on the “margin” of society can develop [41–43]. Likewise, it was observed that marginality was also positively correlated with an increase in anomalous perceptions [4]. Anomalous perceptions are apparently hallucinatory experiences, and those who live them usually experience them as a phenomenon without scientific explanation [44]. Believers tend to interpret anomalous perceptions as the justification that “they have experienced a supernatural phenomenon” [45]. The hypothetical model of social marginality requires analyzing communication systems, access and the quality of information consumption.

Precisely, during the social quarantine, the consumption of information could be characterized by (1) the *disintermediation* between the original information sources and the people-recipients of the respective information [46], 2) the *acceleration of digitalization,* which has facilitated mass access to information and has changed the way of informing oneself about what is happening in reality [47]; and (3), the two previous characteristics contribute to what Innerarity and Colomina (2020) call an *infodemic* or population saturation in the face of so much amount and type of information [48]. At the same time, these three characteristics and the lack of trust in conventional media suggest that the population could have more difficulties in differentiating objective and credible information from pseudoscientific information based on false news [46, 48].

In reality, *social marginality* - originally understood as the personal and geographic isolation of the population - during the quarantine, it has been limited to only *physical* isolation between people, since the acceleration of digitization has allowed individuals with access to technologies, to remain communicated. In other words, the population could suffer various types of “marginalities”, not limited exclusively to the initial idea of “social marginality”. In this line, the quarantine derived from COVID-19 would be related to a “physical-affective” marginality, whose lack of physical contact would have an impact on the management, expression and use of *emotions* [13, 14]. Thus, this type of marginality could be understood as a *physical-affective marginality* that would be different from the social marginality theory”.

Therefore, all the aforementioned involve understanding the social quarantine from three perspectives: (1) should address the psychopathological risks that the social marginality theory warns. According to the social marginality theory, the concept of psychopathological risks should be understood or defined as the tendency to develop attenuated symptoms related to schizoaffective disorders in the general non-clinical population [49]. This expression should not be extrapolated to other mental disorders. (2) The characteristics related to the use and interpretation of the information during quarantine should be taken into account. (3) Finally, the perception of lack of control (related to tolerance to uncertainty) should also be included, which according to the SUB model [4] would explain the development of magical and pseudoscientific beliefs. As determined by the SUB model, pseudoscientific beliefs can be defined as the irrational acceptance (based on magical thinking) of the existence of phenomena that are impossible according to the epistemology of current scientific knowledge [50].

These three points allow characterizing the social quarantine and propose the objectives of this research. Likewise, the definitions of psychopathological risks and pseudoscientific beliefs also represent an operative way of defining variables that are also found in the objectives.

### Objectives

This study aims to analyze the impact of social quarantine during the COVID-19 crisis on magical thinking, pseudoscientific beliefs, anomalous perceptions and psychotic phenotype in subjects from the Spanish general population. The discussion and debates derived from this study are as follows:

1. If one of the characteristics of quarantine is based on social marginality, then the debate raised by this research is based on the following question: How would *physical-affective* marginality affect the levels of magical thinking and pseudoscientific beliefs?
2. If *disintermediation*, the *acceleration of digitization* and the *infodemic* are implicit attributes present in the quarantine, the following debate also arises: Could the probable changes observed in the scores of pseudoscientific beliefs be explained by the three previous characteristics?
3. If the perception of lack of control is one of the causal factors that would justify why pseudoscientific beliefs are developed, then the following question could be discussed: Could *disintermediation*, the *acceleration of digitalization* and the *infodemic* increase the lack of perceived control generating a consecutive increase in pseudoscientific beliefs? For this question, the results should be obtained with significant increases in pseudoscientific beliefs, anomalous perceptions and the psychotic phenotype.

Finally, the study contrasted the following hypothesis: *the levels of pseudoscientific beliefs and anomalous perceptions vary significantly before (pretests) and after (posttests) quarantine due to the effects of “physical-affective marginality”.*

## Materials and methods

### Participants

A total of 99 women and 75 men (174 subjects in total) of legal age (mean = 28.82; standard deviation = 7.943) participated. A total of 41.4% of the participants resided in Madrid, and 58.6% lived in Barcelona. All of them signed a consent form authorizing their voluntary participation. Likewise, they also stated that they had no psychiatric history.

### Instruments

#### Multivariable multiaxial suggestibility inventory − 2 reduced (MMSI-2-R)

It is a self-report questionnaire composed of 49 polytomous items distributed in 6 dimensions or scales: *Visual and Auditory Perception* (Pva); *Cenesthetic Perception* (Pc); *Olfactory Perception* (Po); *Touch Perception* (Pt); *Taste Perception* (Pg); and *Paranoid Experience* (Et). The answers are coded using a Likert scale that fluctuates between 1 and 5. 1 means “strongly disagree” and 5 “strongly agree”. Both versions offer guarantees on their validity and reliability, whose internal consistency indices are greater than 0.8 in all scales [51]. Table 1 reports the description of each dimension and the reliability coefficients.

**Table 1:** Description of MMSI-2-R dimensions and reliability coefficients

AB	Complete denomination	What do the MMSI-2-R scales assess?	Cronbach’s *alpha*	McDonald’s *omega*
Pva	*Visual and Auditory Perception*	Perceptual disturbances whose sensory object is captured visually and auditorily (e.g., seeing ghosts, inexplicable shadows, and hearing voices of deceased beings).	0.987**	0.987**
Pc	*Cenesthetic Perception*	Perceptual disturbances related to depersonalization and derealization (e.g., not recognizing places that are habitual for the patient and experiencing the sensation of leaving one’s own body as an external observer).	0.988**	0.99**
Po	*Olfactory Perception*	Perceptual disturbances whose sensory object is captured through smell (e.g., perceiving odors that other people do not perceive or perceiving odors far from the place where the patient is).	0.984**	0.985**
Pt	*Touch Perception*	Perceptual disturbances whose sensory object is captured using touch or supposed physical contact (e.g., believing that a deceased being has touched you or feeling that something unknown has paralyzed your body).	0.996**	0.996**
Pg	*Taste Perception*	Perceptual changes related to the taste of food (e.g., perceiving more intense flavors than usual or feeling an unpleasant or “rotten” taste in food that is actually in a good condition).	0.983**	0.984**
Et	*Paranoid Experience*	Perceptual disturbances related to the belief that supernatural forces seek to control us (e.g., feel the presence of energies or spirits that want to harm you).	0.949**	0.949**

*AB* abbreviation of the scales’ denomination. Abbreviations do not coincide with the complete denominations as they come from the Spanish version of the MMSI-2-R. ** > 0.8 (reliability coefficients are excellent)

#### Australian sheep-goat scale (ASGS)

It is a brief scale formed by 18 items that examine pseudoscientific beliefs and experiences. Originally, this scale was developed and validated in Australia [52], but A. Escolà-Gascón and L. Storm developed the Spanish adaptation (which has not yet been published), which also shows adequate validity and reliability of the test (Guttman’s *lambda* = 0.93). The responses to the 18 items can be coded in two ways, either complying with the original protocol or the following coding can be applied: 0 = “false”, 1 = “I doubt my answer” and 2 = “true”. This coding was used in the Spanish adaptation and has also been shown to be reliable (McDonald’s *omega* = 0.92) [53]. Given that the Spanish adaptation of the ASGS is not published, the ASGS scale translated into Spanish used in this study is attached to this report (see Supplementary Materials).

#### Community assessment of psychic experiences-42 (CAPE-42)

It is a psychometric scale widely used to evaluate the psychotic phenotype in subjects from the general population [25]. It consists of 3 main dimensions: *Positive Dimension* (hereafter PD) (composed of 20 items), (2) *Negative Dimension* (hereafter ND) (consisting of 14 items), and (3), *Depressive Dimension* (hereafter DD) (contains 8 items). In total, there are 42 items whose responses are quantified following the Likert model with 5 response options. The 1 means “almost never” and the 5 “almost always”. The CAPE-42 was translated and adapted with the Spanish population [54]. This adaptation presents satisfactory reliability indices and construct validity according to the original version of the test. This version was the one used in this study. Table 2 presents a description of each scale and reliability coefficients.

**Table 2:** Description of CAPE-42 dimensions and reliability coefficients

AB	Complete denomination	What do the CAPE-42 scales assess?	Cronbach’s *alpha*
PD	*Positive Dimension*	Analyzes perceptual disturbances and hallucinations expressed in an attenuated and subclinical way (e.g., reading other people’s thoughts).	0.84**
ND	*Negative Dimension*	Analyze clinical symptoms related to difficulties in social and affective relationships (e.g., having the feeling that people do not understand you or difficulties expressing and sharing emotions with others).	0.78*
DD	*Depressive Dimension*	Analyze clinical symptoms related to sudden feelings of sadness and loneliness (this means, without apparent explanation) (e.g., feelings of hopelessness or lack of energy to carry out daily activities).	0.79*

*AB* abbreviation of the scales’ denomination

** > 0.8 (reliability coefficients are excellent), * > 0.7 (reliability coefficients are good)

The subscale that measured the psychopathological impact of psychotic symptoms was not applied because the scales of the CAPE-42 were analyzed as dependent variables (and not as independent variables). The aim was to analyze the impact of the social quarantine derived from COVID-19 on subclinical psychotic symptoms and not vice versa.

### Procedures

In this study, hypothesis contrast tests were applied by comparing means between two repeated samples. The aim was to verify whether social quarantine could alter perceptual processes and magical belief systems.

Initially, the purpose of this research was to replicate the psychometric properties of the MMSI-2-R by examining its convergent validity with respect to the ASGS and CAPE-42 scales. During December 2019 and January, February and March 2020, 346 subjects responded to the questionnaires. When in Spain, the *state of alarm* was decreed on March 14 due to the health crisis caused by COVID-19 [55], the research had to be interrupted to meet other more urgent needs related to this crisis. However, with the state of alarm in Spain, the total social quarantine of the population was also decreed during the following 2 weeks of March. Subsequently, the quarantine lasted until May 10. This fact caused the research team to make a decision regarding how to take advantage of the research sample. Understanding the importance of the scientific and statistical analysis of the social, health and economic impact of the SARS-CoV-2 virus, the research team decided to reorganize the priorities of the original study and made the quick decision to contact the participants again by email to return to answer telematically to the MMSI-2-R, CAPE-42 and ASGS questionnaires. The contact with the participants began on May 11 (also the day in which the first phase began to resolve the quarantine and return to normal social relations). The deadline for receiving the responses was May 21. This decision was made with the aim of adapting the collection of posttests to the circumstances of each participant, since it was not possible for all participants to respond to the questionnaires on the last day of quarantine. Of the 346 subjects, only 174 subjects answered the tests again. In the following week, the data were analyzed, and the present report was written.

### Data analysis

The data were processed in the JASP and JAMOVI programs, both of which are open access and were created by the same research group [56]. *Student’s t-*tests were applied for repeated samples, their nonparametric version (*Wilcoxon* test) and a *Bayesian* estimation were also performed from the *Bayes factor* in favor of the alternative hypothesis (hereafter *BF*_*10*_). The a priori probabilities were adjusted to 50% such that the null hypothesis (*H*_*0*_) and alternative hypothesis were *equiprobable*. The *Cauchy scale* was also adjusted for convenience to 0.707. From the *BFs,* the probability (*P*) that the alternative hypothesis (*H*_*1*_) reproduces the observed data (*D*) could be obtained. The following transformation formula was used:

$$ {BF}_{10}=\frac{P\left(D|{H}_1\right)}{P\left(D|{H}_0\right)}\asymp P\left({H}_1|D\right)=\frac{BF_{10}}{BF_{10}+1} $$

This is possible because the *BF*_*10*_ are *likelihood ratios*, but they differ from the *likelihood quotient* in that the parameters of the previous equation are obtained by integration and not by maximizing. As a complement, measures of effect size were also estimated using *Cohen’s d*. The risk of error was adjusted to 1% in all contrasts and to 5% for the credibility intervals of the Bayesian estimates.

## Results

Table 3 presents the descriptive statistics for the dependent variables used and for each application of the tests (pre- and posttest applications).

**Table 3:** Descriptive statistics for each variable

Scales	Measures	Means	Standard deviation	95% Credible interval^a^	
				Lower	Upper
*Pseudoscientific beliefs*	Pre-test	12.805	8.529	11.528	14.081
	Post test	17.557	6.668	16.560	18.555
*Visual and auditory perception*	Pre-test	35.368	11.398	33.662	37.073
	Post test	36.356	12.253	34.523	38.190
*Touch perception*	Pre-test	17.345	5.291	16.553	18.137
	Post test	21.034	6.582	20.050	22.019
*Olfactory perception*	Pre-test	16.925	4.683	16.225	17.626
	Post test	19.632	5.453	18.816	20.448
*Taste perception*	Pre-test	8.879	2.610	8.489	9.270
	Post test	10.615	3.475	10.095	11.135
*Cenesthetic perception*	Pre-test	25.856	6.978	24.812	26.900
	Post test	31.310	7.564	30.178	32.442
*Paranoid experience*	Pre-test	5.805	1.874	5.524	6.085
	Post test	9.201	2.964	8.758	9.645
*Positive dimension*	Pre-test	28.448	5.006	27.699	29.197
	Post test	31.885	5.758	31.023	32.747
*Negative dimension*	Pre-test	24.862	6.839	23.839	25.885
	Post test	24.460	6.623	23.469	25.451
*Depressive dimension*	Pre-test	14.879	5.003	14.131	15.628
	Post test	24.408	3.983	23.812	25.004

^a^Credible interval was taken from the Bayesian analyses

Increases in the average values can be observed in all dependent variables (except for the *Negative Dimension* scale). To compare whether these increases are significant, different means comparison tests were applied for each variable. This information is shown in Table 4.

**Table 4:** Means compassion using t test, Wilcoxon test and Bayes factors

Scales	*t test* (*p* values)	*Wilcoxon* (*p* values)	*BF*^a^_*10*_ (% error)	*P*(*H*_1_| *D*)	*Cohen’s d*^b^
*Pseudoscientific beliefs*	− 14.172 *p* < 0.001	345.5 *p* < 0.001	3.357e+ 27 ≈ 36.125 (∼0)	0.97306*	− 1.074
*Visual and auditory perception*	−3.014 *p* = 0.003	4503.5 *p* = 0.118	6.603 (2.524%)	0.86847	−0.228
*Touch perception*	−14.382 *p* < 0.001	360.5 *p* < 0.001	1.324e+ 28 ≈ 31.6 (∼0)	0.96933*	−1.090
*Olfactory perception*	−17.382 *p* < 0.001	85.7 *p* < 0.001	2.865e+ 36 ≈ 43.788 (∼0)	0.97767*	−0.986
*Taste perception*	−13.982 *p* < 0.001	157.5 *p* < 0.001	9.861e+ 26 ≈ 52.805 (∼0)	0.98141*	−1.060
*Cenesthetic perception*	−16.596 *p* < 0.001	67 *p* < 0.001	2.006e+ 34 ≈ 39.453 (∼0)	0.97528*	−1.258
*Paranoid experience*	−24.435 *p* < 0.001	179.5 *p* < 0.001	2.867e+ 54 ≈ 61.793 (∼0)	0.98407*	−1.852
*Positive dimension*	−23.022 *p* < 0.001	∼0 *p* < 0.001	1.152e+ 51 ≈ 54.131 (∼0)	0.98186*	−1.745
*Negative dimension*	0.666 *p* = 0.507	6438.5 *p* = 0.678	0.105 (∼0)	0.09502	0.05
*Depressive dimension*	−22.736 *p* < 0.001	50 *p* < 0.001	2.289e+ 50 ≈ 56.222 (∼0)	0.98252*	−1.724

^a^*BF*_*10*_ is Bayes Factor

^b^*Cohen’s d* was applied according t tests

*Evidence in favor of the alternative hypothesis (*BFs* > 10)

Table 4 brings together parametric and nonparametric contrast statistics. In most variables, both the *t-*test and the *Wilcoxon* test offer consistent results and indicate that the average increases are significant, with the exception of the *visual and auditory perception* scales (scale belonging to the MMSI-2-R). and *Negative Dimension* (belonging to the CAPE-42 test), whose critical levels are greater than 0.01. Precisely in the results of Table 4, according to *Cohen’s d* indices, the effects that have a larger or larger size are found for the variables *Cenesthetic Perception*, *Paranoid Experience*, *Positive dimensions* and *Depressive Dimension*. However, the *Pseudoscientific Beliefs* and *Taste Perception* variables also show *Cohen’s d* indices greater than 1 (taken as absolute values).

Attending the *BFs* and the *P*(*H*_1_| *D*), the results also support the statistical decisions specified so far. More specifically, the *BFs* indicate that the alternative hypothesis fits the empirical data between 53 and 61 times more than the null hypothesis for the variables *Taste Perception, Paranoid Experience, Positive Dimension* and *Depressive Dimension*. For the variables whose *BFs* were greater than 10, the distributions were characterized as “a posteriori” based on the Bayesian estimation performed. These distributions allow us to know the *credibility intervals* estimated at 95%. Within the limits of these intervals, the mediated and estimated *Cohen’s d* effect sizes can be located.

The pre- and postscores of each scale were also examined using *Pearson linear correlations*. Table 5 presents the results of this analysis.

**Table 5:** Correlation matrix between variables pre and post-tests

		*Pre-tests*									
		PB	Pva	Pt	Po	Pg	Pc	Et	PD	ND	DD
*Post-tests*	PB	0.858*	0.526*	0.432*	0.227*	0.333*	0.318*	0.547*	0.587*	0.179*	0.213*
	Pva	0.591*	0.936*	0.551*	0.467*	0.613*	0.465*	0.704*	0.4*	0.166	0.216*
	Pt	0.482*	0.579*	0.186*	0.426*	0.543*	0.486*	0.476*	0.38*	0.123	0.25*
	Po	0.374*	0.391*	0.406*	0.929*	0.487*	0.21*	0.392*	0.183*	0.01	0.191*
	Pg	0.299*	0.469*	0.421*	0.563*	0.893*	0.222*	0.412*	0.029	0.105	0.094
	Pc	0.524*	0.677*	0.539*	0.205*	0.404*	0.825*	0.594*	0.496*	0.154	0.263*
	Et	0.606*	0.652*	0.485*	0.381*	0.51*	0.414*	0.804*	0.509*	−0.022	0.054
	PD	0.722*	0.581*	0.45*	0.225*	0.291*	0.38*	0.676*	0.943*	0.081	0.291*
	ND	0.172	0.252*	0.214*	0.171	0.19*	0.107	0.133	0.211*	0.299*	0.354*
	DD	0.254*	0.286*	0.3*	−0.031	0.031	0.155	0.298*	0.358*	0.236*	0.259*

*PB* Pseudoscientific beliefs, *Pva* Visual and Auditory Perception, *Pt* Touch Perception, *Po* Olfactory Perception, *Pg* Taste Perception, *Pc* Cenesthetic Perception, *Et* Paranoid Experiences, *PD* Positive Dimension, *ND* Negative Dimension, *DD* Depressive Dimension

*p < 0.01

The weight of the correlations increases as the size of the effects is larger. This seems to coincide with previous results. The only value of the matrix trace in Table 5 that yields an incoherent weight with the effect size obtained in Table 4 is that belonging to the *Depressive Dimension* scale. This suggests that the changes observed in this variable and specifically in the posttest tend to independence in relation to the measures applied before quarantine.

As a joint decision, given the results obtained, the null hypothesis can be rejected and the alternative maintained, which supports the relationship between social quarantine and significant increases for all scales (except for the Pva and ND dimensions).

## Discussion

In this study, we wanted to verify the effects of the social and health consequences of social quarantine on the variables pseudoscientific beliefs, anomalous perceptions and the traits that describe the psychotic phenotype. The contrast tests applied reveal that the scores in these variables increase after 57 days of social quarantine.

### Interpretation of the results

The hypothetical social marginality theory related to pseudoscientific beliefs has rarely been investigated outside the experimental framework [41, 42]. In reality, social marginality was studied from a sociocultural perspective limited to geographically isolated regions, whose living conditions differed from the normative lifestyle of large Western cities (e.g., towns with few inhabitants or villages located in climatologically aversive environments) [42]. Unlike the geographically isolated areas, the social quarantine during the COVID-19 crisis was only physical since technologies allowed us to maintain communications and digitize human relations.

Taking as a reference the results obtained, it can be concluded that social quarantine increases levels of magical thinking, pseudoscientific beliefs and anomalous perceptions. However, knowing that this research is not purely experimental, if one were to consider why these increases occur, hypothetical inferences should be made related to the sociosanitary characteristics implicit in the quarantine. As already mentioned, these characteristics may be related to psychological and psychopathological variables as well as to other variables associated with communication and access to information. From here, the following is proposed: is it possible that the *disintermediation*, the *acceleration of digitization* and the *infodemic* - especially the latter - can alter the way of interpreting information by the population generating generalized *fatigue* and a *saturation of* stimuli? Is it possible that fatigue and saturation are the mediating variables responsible for this increase? If the results of this research indicate that magical thinking has increased, so can *false news*, disinformation and *pseudoscientific* information. Then, as some international studies point out, it is possible that disinformation may be another of the causal variables of these increases [57, 58]. It is noted that the previous questions would not be justified if the scores on the *Paranoid Experiences* scale had not obtained the highest effect size. It is important to stop at this point because this scale warns that the levels of distrust and paranoia are those that have increased the most (with respect to the other psychological indicators evaluated). To the team’s surprise, this increase coincides with the results published by the CAC (*Consell de l’Audiovisual de Catalunya*) on the increase in disinformation and false news during the quarantine derived from COVID-19 (whose rates reach 80%) [59].

As seen, these issues are merely speculative and invite future research to correlate the data related to false news publications with the recorded increases in magical thinking and pseudoscientific beliefs in this research. For this reason, the raw data of the project are available in the file Raw_data_1; thus, other investigations could also be used.

Returning to the characteristics or psychopathological risks related to quarantine, another relevant interpretation falls on the following question: Why in some variables are the sizes of the increase in scores higher than in others? On the one hand, in the case of the Pc scale of the MMSI-2-R, it should be taken into account that kinesthetic perceptions describe alterations related to *depersonalization* and *derealization* processes. Another of the characteristics of quarantine is that the subject had to remain locked up a number of hours higher than usual in limited and nonvariable spaces. That is, in addition to being confined, another characteristic of the quarantine space is that in most cases, it is the same and does not change, although the subject does change activities and tasks throughout the day. The fusion of these two implicit characteristics during periods of quarantine could generate states of confusion in the subject that would trigger kinesthesia as the main perceptual alteration. On the other hand, the psychotic phenotype is still a subclinical marker relative to the risks of suffering future psychotic symptoms. The fact that PD and DD (belonging to the CAPE-42 scale) have also shown significant increases indicates that quarantine could increase risk levels in suffering from future psychotic behaviors. The PD scale examines psychotic hallucinations, and its effects are the second highest (see *Cohen’s d* in Table 4). These data - integrating it with the results of the Pc scale of the MMSI-2-R - warns that the hallucinatory pictures could increase after the subjects experience prolonged states of quarantine and, specifically, that the increase is observed in the kinesthetic-type hallucinatory contents.

If scientific research should have professional applicability and social influence, then the questions that have been posed should help the respective media and interested agencies to consider what control should be exercised over information traffic and disinformation in the crisis stages. It is precisely in these periods when people have greater psychopathological risks (see the results in Table 4) and are more vulnerable to suffering the negative consequences of disinformation and false news (see data published by the CAC), associated with effects of the state of social quarantine itself. In addition, taking into account what has been discussed, magical beliefs could also be altered by the way in which information is consumed, accessed and interpreted. The sense of control that they can transmit to the believing subject (see the SUB model proposed by Irwin) [4] could justify its implementation and activation, but its increase is also conditioned on the dissemination and manipulation of information. How to analyze the consumption of information and ensure its credibility is one of the challenges that can be posed based on the results obtained and based on the COVID-19 crisis.

### Criticisms and limitations

The limitations of this study can be summarized in six key points:

1. The applied design was not experimental. This means that the impact of quarantine cannot be interpreted in absolute causal terms. It is for this reason that “conditional” arguments have been used in the analysis and speculation with the results obtained. The findings of this study support that there may be a causal relationship between the state of quarantine and changes in the behaviors examined, but this causality has not been contrasted. Therefore, this should be replicated in the future to optimize both internal and external validity.
2. The pretests were performed by the subjects on excessively heterogeneous dates before starting quarantine (between the first subject who responded to the pretests and the last subject before the onset of the state of alarm, 46 days passed). How this variability associated with pretest dates could have affected is something that could not be controlled in this study and will not be controlled, since it is not possible to know the factors that intervened in the lives of the subjects of the sample during those 46 days. One possible solution that was considered was the exclusion of subjects who had answered the pretests before February 29 (15 days before the state of alarm); in this way, the effect of the variability relative to the dates could have been reduced. The problem with this methodological decision is that it would excessively damage the external validity of the results, since the sample would be reduced to less than 30 subjects (a critical number for hypothesis testing). Therefore, a design was chosen that would benefit external validity (facilitating the increase in sample size and the replicability of the results). Likewise, in Spain, the COVID-19 health crisis was reported by the media almost suddenly and with no time frame to act, prevent and make quick decisions that would allow for the implementation of a necessary and complex study such as the one presented here.
3. The fact that pseudoscientific beliefs have increased after quarantine does not mean that this increase is psychopathological in itself. Nor does it mean that the increase is explained by the “psychotic phenotype” (unlike the PD scale of the CAPE-42). Taking into account the *Scientific Unexplained Beliefs Model* [4], pseudoscientific beliefs may have increased due to uncertainty and the feeling of lack of control and not so much due to the presence of subclinical psychotic mechanisms in the individual. The increase in the risk indices evaluated by the CAPE-42 and the increase in pseudoscientific beliefs may be correlative in the sample used but does not imply that one group of variables causally justifies the increase in the others. This is important to note, since the fact of having divergent beliefs should not be confused with the ontological principles of science (e.g., beliefs in the “supernatural”) and the possibility of suffering a dissociative-psychotic picture.
4. In this research, no indicators were recorded in the posttests that would allow knowing the compliance and management of the state of quarantine of each subject. All participants declared having met quarantine (which was the basic condition and sufficient to perform the posttests). In addition, the control or record of the behavioral indicators on how the participant complied with the quarantine represents an object outside this research: the impact of the quarantine was limited to the specified dependent variables. However, it is true that such information would have made possible the inclusion of new independent variables that would interact with the main variable pre- and posttests. To what extent the latter would improve and optimize the already made contrasts is something that is unknown.
5. Some limitations related to the lack of representativeness of the sample should be discussed. First, sample selection was not probabilistic and could not be weighted according to stratification or cluster selection techniques. This makes it difficult to generalize the findings to the population as a whole. Therefore, it is proposed to interpret the results of this research as a warning and not as a confirmation in statistical terms of the effects of social quarantine on the non-cynical population. Second, although the extrapolation of the results is not completely generalizable, the data and interpretations can be used to rationally and empirically support future research that contrasts similar variables. Specifically, it is recommended to consider sociodemographic markers that provide information on which social groups are most vulnerable to COVID-19. For example, the following question should be addressed: do the elderly (as the most vulnerable social group according to age) tend to develop more or less irrational behaviors than the young people?
6. Finally, the results of the investigation were interpreted in relation to the consumption of information and digital media. However, although data from Spanish public entities were used [59], explicit measurements of these variables were not included in the investigation. Taking into account that the dissemination of pseudoscientific information can lead the population to make bad decisions [60–62], it seems necessary for future studies to relate the degree to which decisions based on pseudoscience increase psychopathological risks. To carry out this analysis, the consumption of pseudoscientific information must be measured. Despite this limitation, the results of this report warn that the effects of pseudoscientific information were involved during the social quarantine, as pseudoscientific beliefs increased in post-tests.

## Conclusions

This research and its results allow us to reach the following conclusions:

1. Understanding that in large cities, the quarantine of the population in their homes has so far represented one of the circumstances closest to the idea of “social marginality”, the results of this research support the extrapolation of the hypothesis of social marginality to a physical-affective level, applied specifically to subjects residing in large cities.
2. The increases in pseudoscientific beliefs, anomalous experiences and even the psychotic phenotype were observable and significant after 57 days of state of alarm and social quarantine. It is concluded that depressive symptoms, psychotic hallucinations, kinesthetic alterations and paranoid experiences were the variables with the largest effect sizes. The Bayesian estimation indicated that the perceptual visual-auditory anomalies (Pva scale of the MMSI-2-R) did not present significant changes; therefore, it does not seem to be a perceptual alteration that is affected by social quarantine. The same happened with the negative symptoms of the ND scale (present in certain psychotic pictures); quarantine had no effect on this variable.
3. It is concluded that the risk of suffering from paranoid, psychotic or dissociative states can easily increase after these days of physical-social isolation. This would also put at risk the mental health of people and would emphasize the urgency of the psychiatric and psychological measures that the legislation and the government should take to protect the most vulnerable medical-psychological profiles in terms of the development of psychotic pictures.
4. As a final conclusion, knowing that the states of paranoia were the experiences that increased the most after the social quarantine, it is worth considering the possibility that an excess of information and disinformation in digital media is one of the variables causing the increases observed for generating confusion and preventing the general population from effectively discriminating between credible information sources and pseudoscientific information sources.

## Supplementary information

**Additional file 1.**

**Additional file 2.**

## Data Availability

All data generated or analyzed during this study are included in this published article (see Raw_data_1) [and its supplementary information files].
