# Supplementary material for: Pseudoscientific beliefs and psychopathological risks increase after COVID-19 social quarantine
Source: Global Health. 2020 Jul 30;16:72. doi: 10.1186/s12992-020-00603-1 (PMC7391050; doi:10.1186/s12992-020-00603-1)
Supplement: Supplementary file 2 — Additional file 2. [file 12992_2020_603_MOESM2_ESM.jasp › index.html]

JASP 


# Results

## Paired Samples T-Test

| Paired Samples T-Test | | | | | | | | | | | | | | | |
| --- | --- | --- | --- | --- | --- | --- | --- | --- | --- | --- | --- | --- | --- | --- | --- |
|  | |  | |  | | Test | | Statistic | | df | | p | | Effect Size | |
| Level\_paranormal\_beliefs\_PRE |  | - |  | Level\_paranormal\_beliefs\_POST |  | Student |  | -14.171 |  | 173 |  | < .001 |  | -1.074 |  |
|  |  |  |  |  |  | Wilcoxon |  | 345.500 |  |  |  | < .001 |  | -0.946 |  |
| Level\_MMSI\_R\_Pva\_PRE |  | - |  | Level\_MMSI\_R\_Pva\_POST |  | Student |  | -3.014 |  | 173 |  | 0.003 |  | -0.228 |  |
|  |  |  |  |  |  | Wilcoxon |  | 4503.500 |  |  |  | 0.118 |  | -0.149 |  |
| Level\_MMSI\_R\_Pt\_PRE |  | - |  | Level\_MMSI\_R\_Pt\_POST |  | Student |  | -14.382 |  | 173 |  | < .001 |  | -1.090 |  |
|  |  |  |  |  |  | Wilcoxon |  | 360.500 |  |  |  | < .001 |  | -0.945 |  |
| Level\_MMSI\_R\_Po\_PRE |  | - |  | Level\_MMSI\_R\_Po\_POST |  | Student |  | -17.382 |  | 173 |  | < .001 |  | -1.318 |  |
|  |  |  |  |  |  | Wilcoxon |  | 87.500 |  |  |  | < .001 |  | -0.986 |  |
| Level\_MMSI\_R\_Pg\_PRE |  | - |  | Level\_MMSI\_R\_Pg\_POST |  | Student |  | -13.982 |  | 173 |  | < .001 |  | -1.060 |  |
|  |  |  |  |  |  | Wilcoxon |  | 157.500 |  |  |  | < .001 |  | -0.962 |  |
| Level\_MMSI\_R\_Pc\_PRE |  | - |  | Level\_MMSI\_R\_Pc\_POST |  | Student |  | -16.596 |  | 173 |  | < .001 |  | -1.258 |  |
|  |  |  |  |  |  | Wilcoxon |  | 67.000 |  |  |  | < .001 |  | -0.990 |  |
| Level\_MMSI\_R\_Et\_PRE |  | - |  | Level\_MMSI\_R\_ET\_POST |  | Student |  | -24.435 |  | 173 |  | < .001 |  | -1.852 |  |
|  |  |  |  |  |  | Wilcoxon |  | 179.500 |  |  |  | < .001 |  | -0.974 |  |
| CAPE42\_1\_HAL\_PRE |  | - |  | CAPE42\_1\_HAL\_POST |  | Student |  | -23.022 |  | 173 |  | < .001 |  | -1.745 |  |
|  |  |  |  |  |  | Wilcoxon |  | 0.000 |  |  |  | < .001 |  | -1.000 |  |
| CAPE42\_2\_NEGATIVE\_PRE |  | - |  | CAPE42\_2\_NEGATIVE\_POST |  | Student |  | 0.666 |  | 173 |  | 0.507 |  | 0.050 |  |
|  |  |  |  |  |  | Wilcoxon |  | 6438.500 |  |  |  | 0.678 |  | 0.038 |  |
| CAPE42\_3\_DEP\_PRE |  | - |  | CAPE42\_3\_DEP\_POST |  | Student |  | -22.736 |  | 173 |  | < .001 |  | -1.724 |  |
|  |  |  |  |  |  | Wilcoxon |  | 50.000 |  |  |  | < .001 |  | -0.993 |  |
|  | | | | | | | | | | | | | | | |
|  |  |  |  |  |  |  |  |  |  |  |  |  |  |  |  |
| --- | --- | --- | --- | --- | --- | --- | --- | --- | --- | --- | --- | --- | --- | --- | --- |
| *Note.*  For the Student t-test, effect size is given by Cohen's *d* ; for the Wilcoxon test, effect size is given by the matched rank biserial correlation. | | | | | | | | | | | | | | | |

### Assumption Checks

| Test of Normality (Shapiro-Wilk) | | | | | | | | | |
| --- | --- | --- | --- | --- | --- | --- | --- | --- | --- |
|  | |  | |  | | W | | p | |
| Level\_paranormal\_beliefs\_PRE |  | - |  | Level\_paranormal\_beliefs\_POST |  | 0.946 |  | < .001 |  |
| Level\_MMSI\_R\_Pva\_PRE |  | - |  | Level\_MMSI\_R\_Pva\_POST |  | 0.895 |  | < .001 |  |
| Level\_MMSI\_R\_Pt\_PRE |  | - |  | Level\_MMSI\_R\_Pt\_POST |  | 0.891 |  | < .001 |  |
| Level\_MMSI\_R\_Po\_PRE |  | - |  | Level\_MMSI\_R\_Po\_POST |  | 0.924 |  | < .001 |  |
| Level\_MMSI\_R\_Pg\_PRE |  | - |  | Level\_MMSI\_R\_Pg\_POST |  | 0.916 |  | < .001 |  |
| Level\_MMSI\_R\_Pc\_PRE |  | - |  | Level\_MMSI\_R\_Pc\_POST |  | 0.937 |  | < .001 |  |
| Level\_MMSI\_R\_Et\_PRE |  | - |  | Level\_MMSI\_R\_ET\_POST |  | 0.792 |  | < .001 |  |
| CAPE42\_1\_HAL\_PRE |  | - |  | CAPE42\_1\_HAL\_POST |  | 0.889 |  | < .001 |  |
| CAPE42\_2\_NEGATIVE\_PRE |  | - |  | CAPE42\_2\_NEGATIVE\_POST |  | 0.954 |  | < .001 |  |
| CAPE42\_3\_DEP\_PRE |  | - |  | CAPE42\_3\_DEP\_POST |  | 0.973 |  | 0.002 |  |
|  | | | | | | | | | |
|  |  |  |  |  |  |  |  |  |  |
| --- | --- | --- | --- | --- | --- | --- | --- | --- | --- |
| *Note.*  Significant results suggest a deviation from normality. | | | | | | | | | |

### Descriptives

| Descriptives | | | | | | | | | |
| --- | --- | --- | --- | --- | --- | --- | --- | --- | --- |
|  | | N | | Mean | | SD | | SE | |
| Level\_paranormal\_beliefs\_PRE |  | 174 |  | 12.805 |  | 8.529 |  | 0.647 |  |
| Level\_paranormal\_beliefs\_POST |  | 174 |  | 17.557 |  | 6.668 |  | 0.505 |  |
| Level\_MMSI\_R\_Pva\_PRE |  | 174 |  | 35.368 |  | 11.398 |  | 0.864 |  |
| Level\_MMSI\_R\_Pva\_POST |  | 174 |  | 36.356 |  | 12.253 |  | 0.929 |  |
| Level\_MMSI\_R\_Pt\_PRE |  | 174 |  | 17.345 |  | 5.291 |  | 0.401 |  |
| Level\_MMSI\_R\_Pt\_POST |  | 174 |  | 21.034 |  | 6.582 |  | 0.499 |  |
| Level\_MMSI\_R\_Po\_PRE |  | 174 |  | 16.925 |  | 4.683 |  | 0.355 |  |
| Level\_MMSI\_R\_Po\_POST |  | 174 |  | 19.632 |  | 5.453 |  | 0.413 |  |
| Level\_MMSI\_R\_Pg\_PRE |  | 174 |  | 8.879 |  | 2.610 |  | 0.198 |  |
| Level\_MMSI\_R\_Pg\_POST |  | 174 |  | 10.615 |  | 3.475 |  | 0.263 |  |
| Level\_MMSI\_R\_Pc\_PRE |  | 174 |  | 25.856 |  | 6.978 |  | 0.529 |  |
| Level\_MMSI\_R\_Pc\_POST |  | 174 |  | 31.310 |  | 7.564 |  | 0.573 |  |
| Level\_MMSI\_R\_Et\_PRE |  | 174 |  | 5.805 |  | 1.874 |  | 0.142 |  |
| Level\_MMSI\_R\_ET\_POST |  | 174 |  | 9.201 |  | 2.964 |  | 0.225 |  |
| CAPE42\_1\_HAL\_PRE |  | 174 |  | 28.448 |  | 5.006 |  | 0.379 |  |
| CAPE42\_1\_HAL\_POST |  | 174 |  | 31.885 |  | 5.758 |  | 0.437 |  |
| CAPE42\_2\_NEGATIVE\_PRE |  | 174 |  | 24.862 |  | 6.839 |  | 0.519 |  |
| CAPE42\_2\_NEGATIVE\_POST |  | 174 |  | 24.460 |  | 6.623 |  | 0.502 |  |
| CAPE42\_3\_DEP\_PRE |  | 174 |  | 14.879 |  | 5.003 |  | 0.379 |  |
| CAPE42\_3\_DEP\_POST |  | 174 |  | 24.408 |  | 3.983 |  | 0.302 |  |
|  | | | | | | | | | |

#### Descriptives Plots

##### Level\_paranormal\_beliefs\_PRE - Level\_paranormal\_beliefs\_POST

##### Level\_MMSI\_R\_Pva\_PRE - Level\_MMSI\_R\_Pva\_POST

##### Level\_MMSI\_R\_Pt\_PRE - Level\_MMSI\_R\_Pt\_POST

##### Level\_MMSI\_R\_Po\_PRE - Level\_MMSI\_R\_Po\_POST

##### Level\_MMSI\_R\_Pg\_PRE - Level\_MMSI\_R\_Pg\_POST

##### Level\_MMSI\_R\_Pc\_PRE - Level\_MMSI\_R\_Pc\_POST

##### Level\_MMSI\_R\_Et\_PRE - Level\_MMSI\_R\_ET\_POST

##### CAPE42\_1\_HAL\_PRE - CAPE42\_1\_HAL\_POST

##### CAPE42\_2\_NEGATIVE\_PRE - CAPE42\_2\_NEGATIVE\_POST

##### CAPE42\_3\_DEP\_PRE - CAPE42\_3\_DEP\_POST

## Bayesian Paired Samples T-Test

| Bayesian Paired Samples T-Test | | | | | | | | | |
| --- | --- | --- | --- | --- | --- | --- | --- | --- | --- |
|  | |  | |  | | BF₁₀ | | error % | |
| Level\_paranormal\_beliefs\_PRE |  | - |  | Level\_paranormal\_beliefs\_POST |  | 3.357e +27 |  | 2.347e -33 |  |
| Level\_MMSI\_R\_Pva\_PRE |  | - |  | Level\_MMSI\_R\_Pva\_POST |  | 6.603 |  | 2.768e  -5 |  |
| Level\_MMSI\_R\_Pt\_PRE |  | - |  | Level\_MMSI\_R\_Pt\_POST |  | 1.324e +28 |  | 2.529e -32 |  |
| Level\_MMSI\_R\_Po\_PRE |  | - |  | Level\_MMSI\_R\_Po\_POST |  | 2.865e +36 |  | 5.450e -42 |  |
| Level\_MMSI\_R\_Pg\_PRE |  | - |  | Level\_MMSI\_R\_Pg\_POST |  | 9.861e +26 |  | 6.556e -33 |  |
| Level\_MMSI\_R\_Pc\_PRE |  | - |  | Level\_MMSI\_R\_Pc\_POST |  | 2.006e +34 |  | 8.933e -40 |  |
| Level\_MMSI\_R\_Et\_PRE |  | - |  | Level\_MMSI\_R\_ET\_POST |  | 2.867e +54 |  | 2.822e -60 |  |
| CAPE42\_1\_HAL\_PRE |  | - |  | CAPE42\_1\_HAL\_POST |  | 1.152e +51 |  | 8.478e -57 |  |
| CAPE42\_2\_NEGATIVE\_PRE |  | - |  | CAPE42\_2\_NEGATIVE\_POST |  | 0.105 |  | 0.002 |  |
| CAPE42\_3\_DEP\_PRE |  | - |  | CAPE42\_3\_DEP\_POST |  | 2.289e +50 |  | 4.118e -56 |  |
|  | | | | | | | | | |

### Inferential Plots

#### Level\_paranormal\_beliefs\_PRE - Level\_paranormal\_beliefs\_POST

##### Prior and Posterior

#### Level\_MMSI\_R\_Pva\_PRE - Level\_MMSI\_R\_Pva\_POST

##### Prior and Posterior

#### Level\_MMSI\_R\_Pt\_PRE - Level\_MMSI\_R\_Pt\_POST

##### Prior and Posterior

#### Level\_MMSI\_R\_Po\_PRE - Level\_MMSI\_R\_Po\_POST

##### Prior and Posterior

#### Level\_MMSI\_R\_Pg\_PRE - Level\_MMSI\_R\_Pg\_POST

##### Prior and Posterior

#### Level\_MMSI\_R\_Pc\_PRE - Level\_MMSI\_R\_Pc\_POST

##### Prior and Posterior

#### Level\_MMSI\_R\_Et\_PRE - Level\_MMSI\_R\_ET\_POST

##### Prior and Posterior

#### CAPE42\_1\_HAL\_PRE - CAPE42\_1\_HAL\_POST

##### Prior and Posterior

#### CAPE42\_2\_NEGATIVE\_PRE - CAPE42\_2\_NEGATIVE\_POST

##### Prior and Posterior

#### CAPE42\_3\_DEP\_PRE - CAPE42\_3\_DEP\_POST

##### Prior and Posterior

### Descriptives Plots

#### Level\_paranormal\_beliefs\_PRE - Level\_paranormal\_beliefs\_POST

#### Level\_MMSI\_R\_Pva\_PRE - Level\_MMSI\_R\_Pva\_POST

#### Level\_MMSI\_R\_Pt\_PRE - Level\_MMSI\_R\_Pt\_POST

#### Level\_MMSI\_R\_Po\_PRE - Level\_MMSI\_R\_Po\_POST

#### Level\_MMSI\_R\_Pg\_PRE - Level\_MMSI\_R\_Pg\_POST

#### Level\_MMSI\_R\_Pc\_PRE - Level\_MMSI\_R\_Pc\_POST

#### Level\_MMSI\_R\_Et\_PRE - Level\_MMSI\_R\_ET\_POST

#### CAPE42\_1\_HAL\_PRE - CAPE42\_1\_HAL\_POST

#### CAPE42\_2\_NEGATIVE\_PRE - CAPE42\_2\_NEGATIVE\_POST

#### CAPE42\_3\_DEP\_PRE - CAPE42\_3\_DEP\_POST
