# Supplementary figures and images for: Pseudoscientific beliefs and psychopathological risks increase after COVID-19 social quarantine
Source: Global Health. 2020 Jul 30;16:72. doi: 10.1186/s12992-020-00603-1 (PMC7391050; doi:10.1186/s12992-020-00603-1)

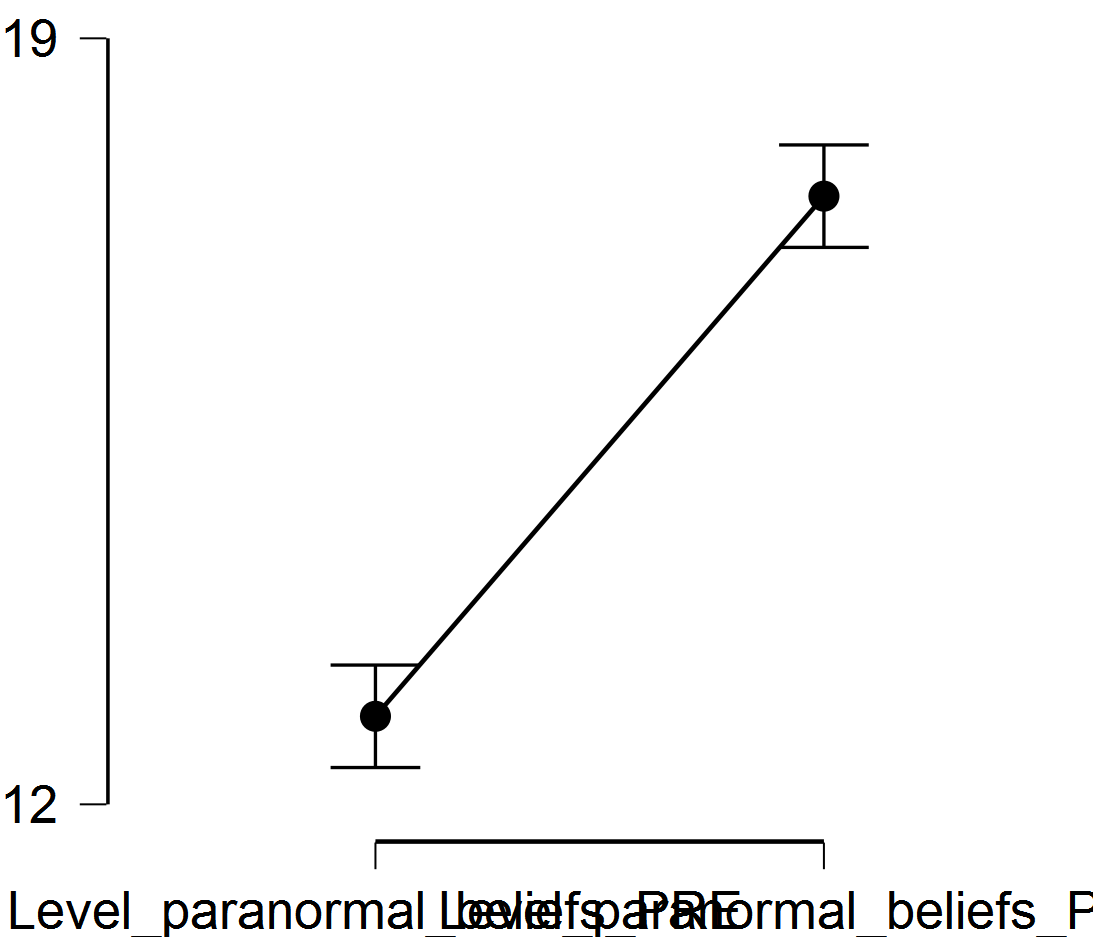

Supplement: Supplementary file 2 — Additional file 2. [file 12992_2020_603_MOESM2_ESM.jasp › resources/0/_20.png]

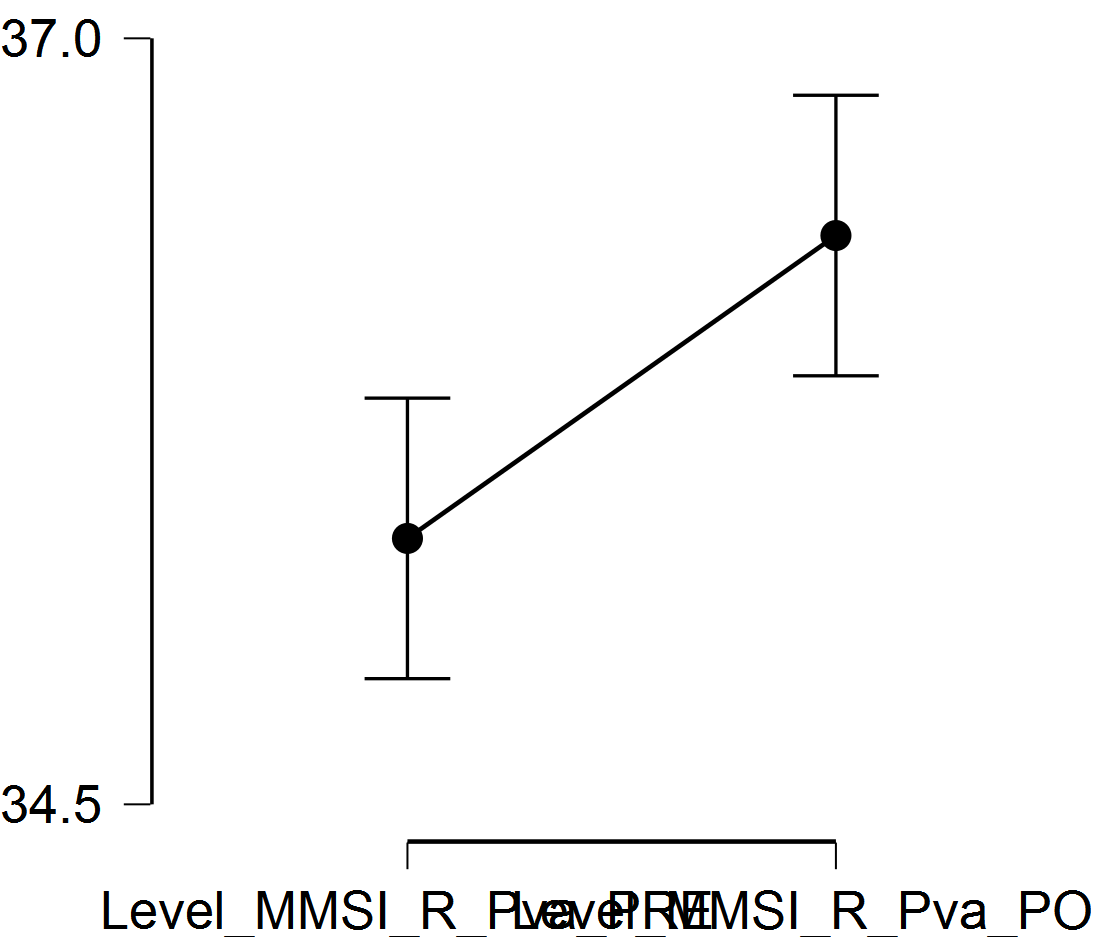

Supplement: Supplementary file 2 — Additional file 2. [file 12992_2020_603_MOESM2_ESM.jasp › resources/0/_21.png]

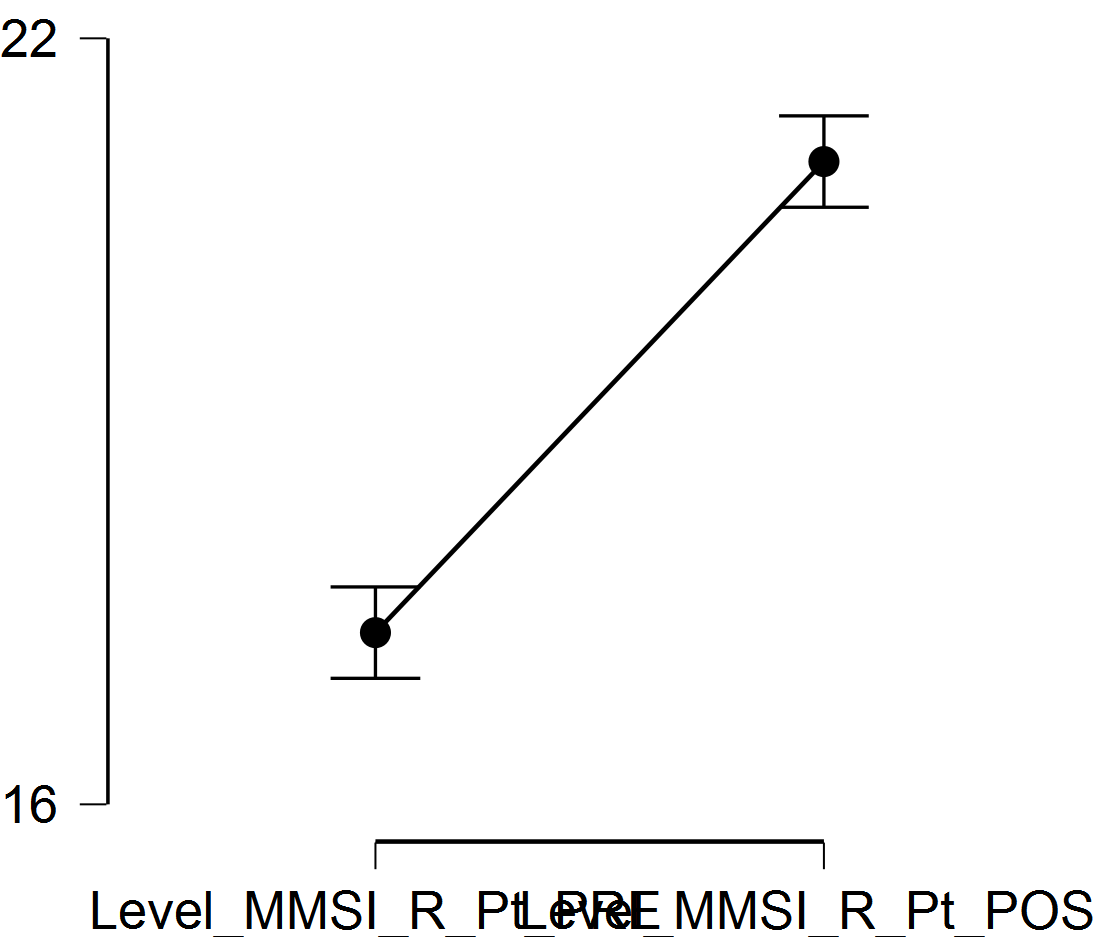

Supplement: Supplementary file 2 — Additional file 2. [file 12992_2020_603_MOESM2_ESM.jasp › resources/0/_22.png]

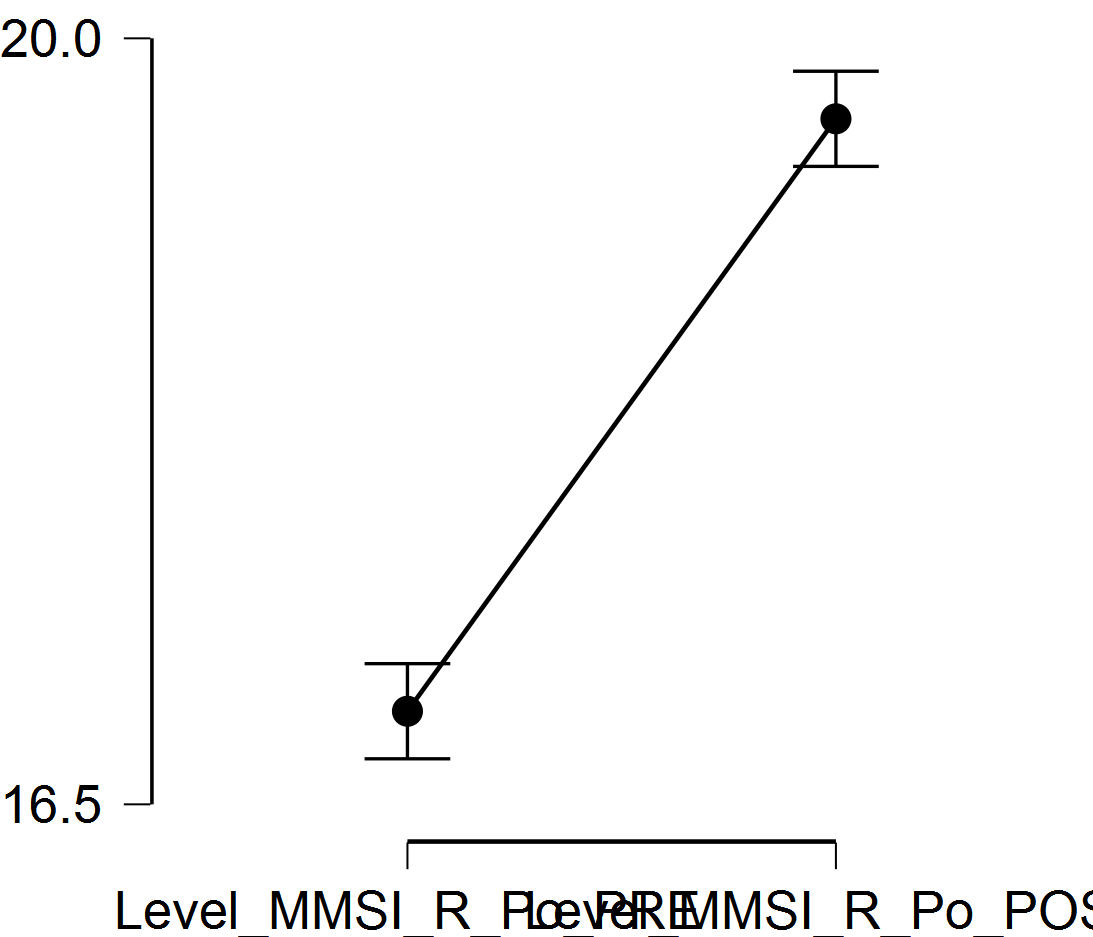

Supplement: Supplementary file 2 — Additional file 2. [file 12992_2020_603_MOESM2_ESM.jasp › resources/0/_23.png]

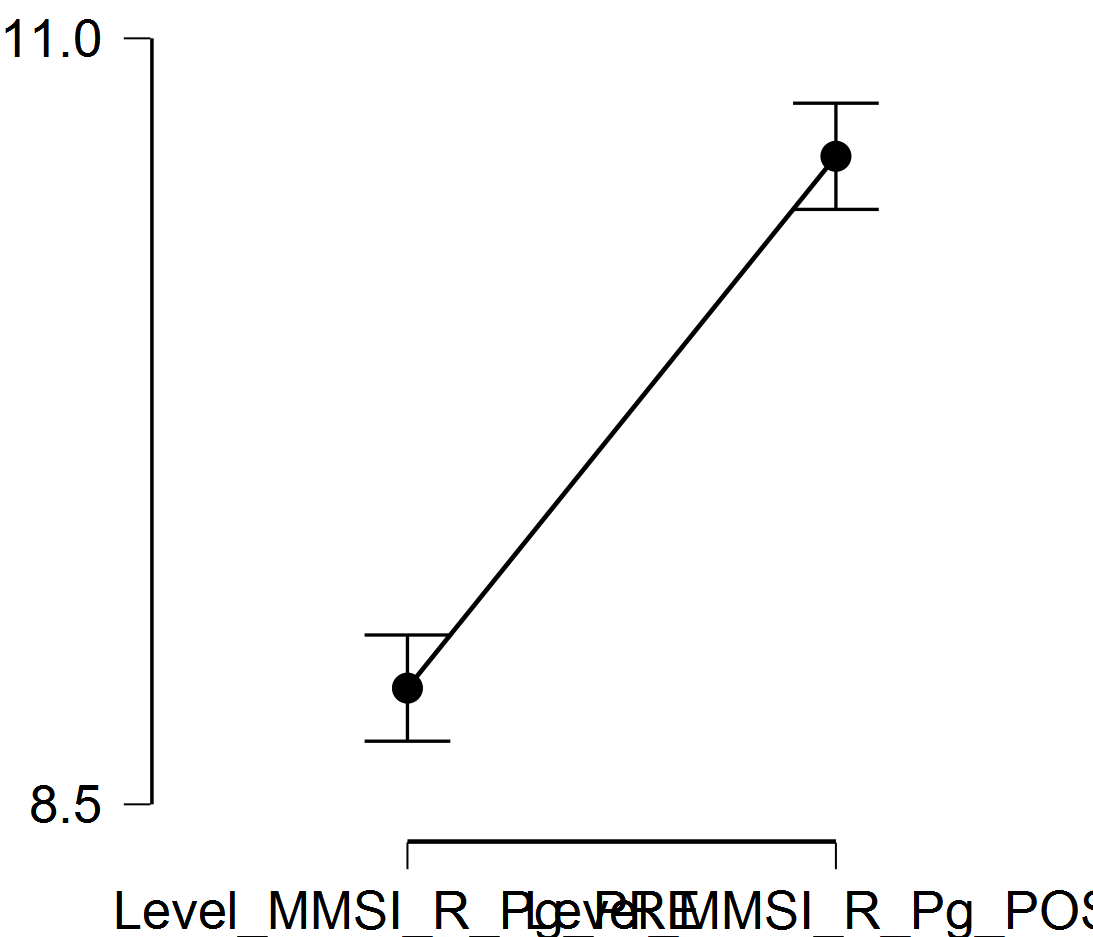

Supplement: Supplementary file 2 — Additional file 2. [file 12992_2020_603_MOESM2_ESM.jasp › resources/0/_24.png]

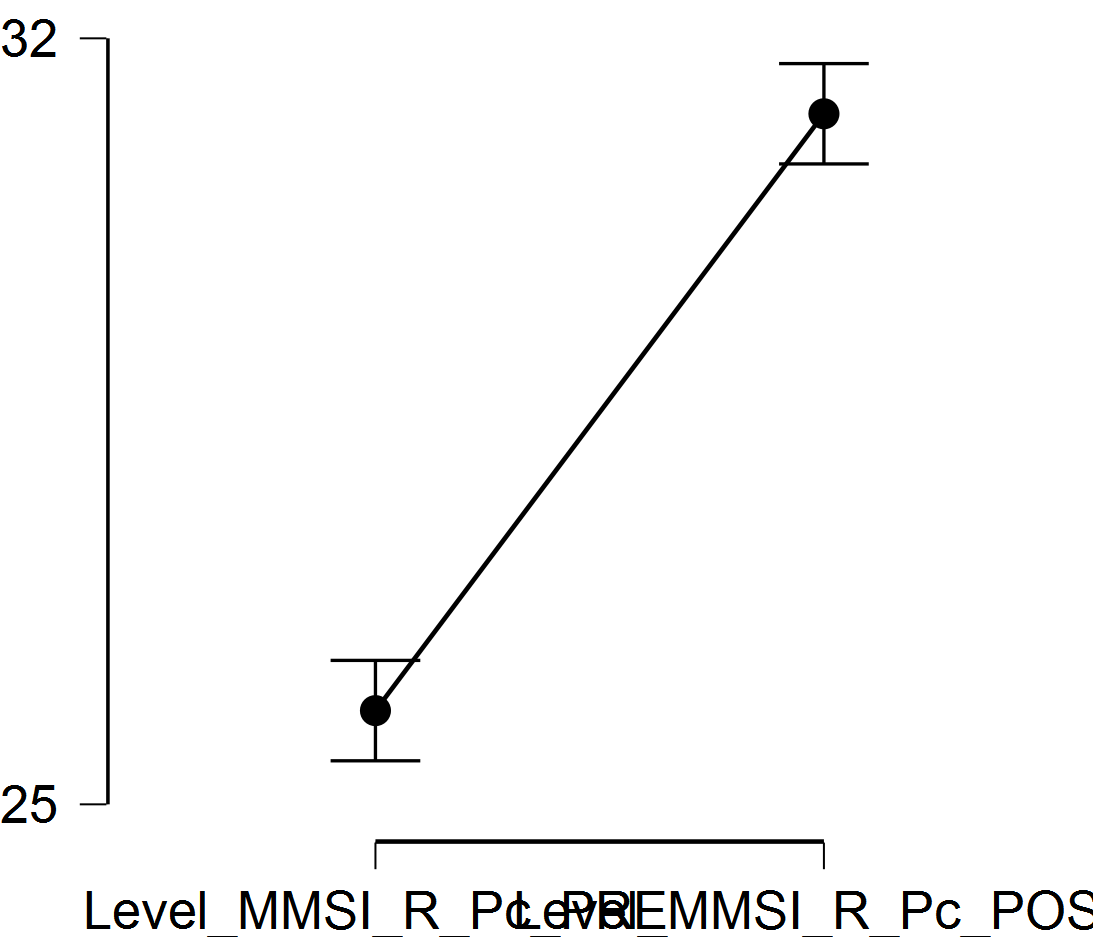

Supplement: Supplementary file 2 — Additional file 2. [file 12992_2020_603_MOESM2_ESM.jasp › resources/0/_25.png]

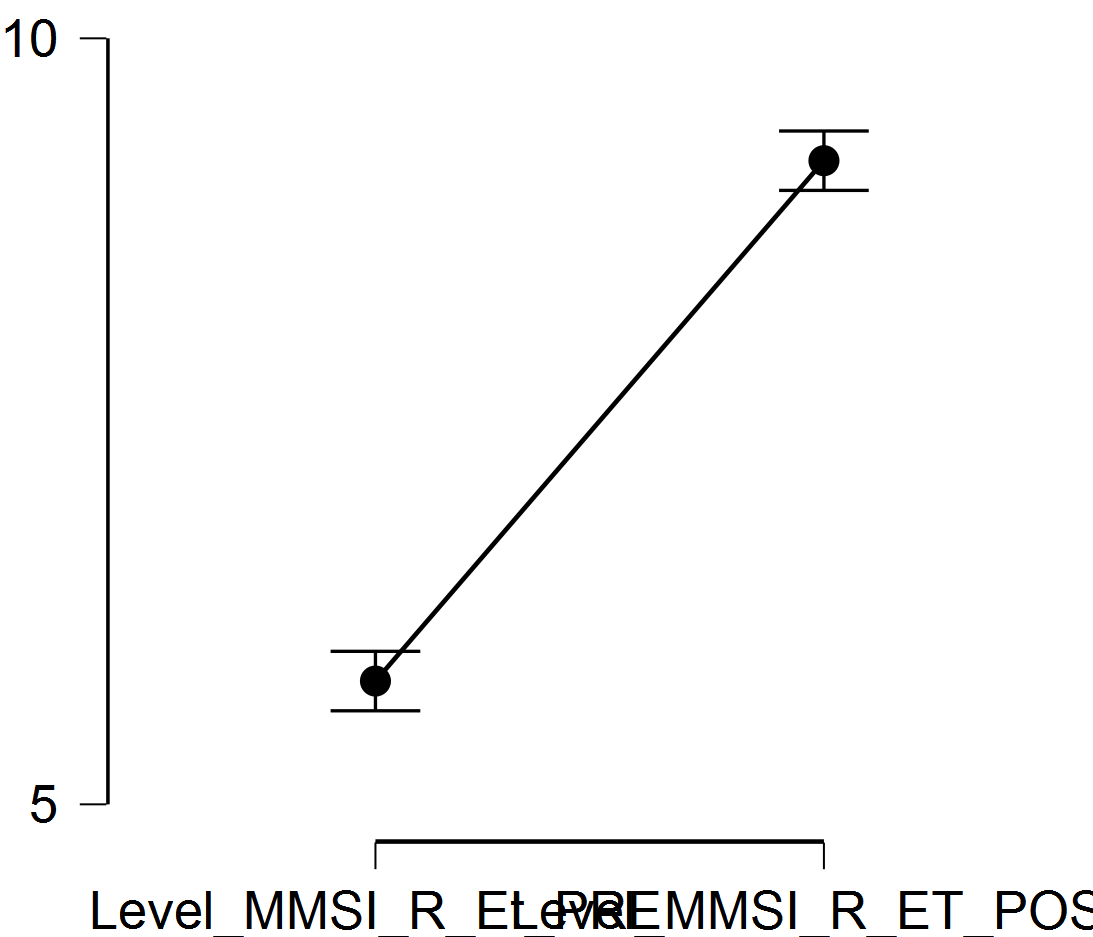

Supplement: Supplementary file 2 — Additional file 2. [file 12992_2020_603_MOESM2_ESM.jasp › resources/0/_26.png]

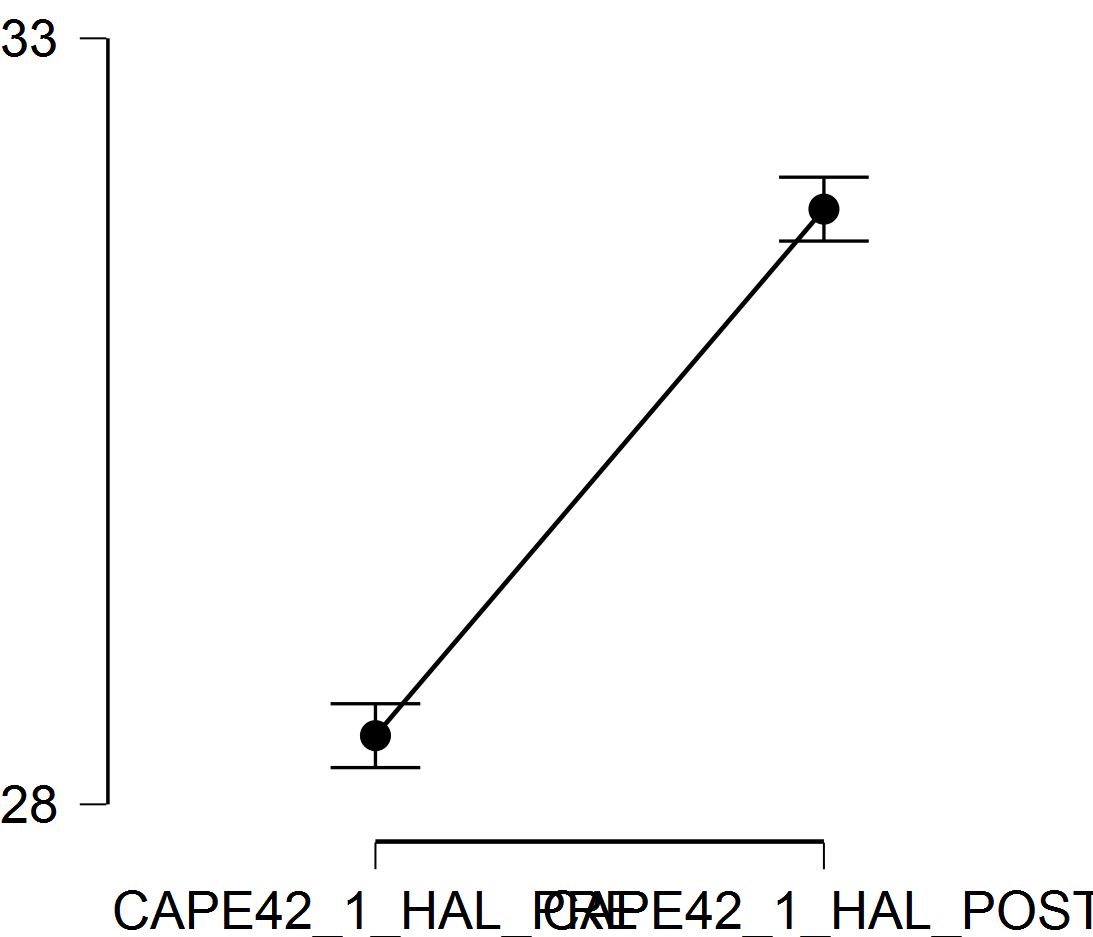

Supplement: Supplementary file 2 — Additional file 2. [file 12992_2020_603_MOESM2_ESM.jasp › resources/0/_27.png]

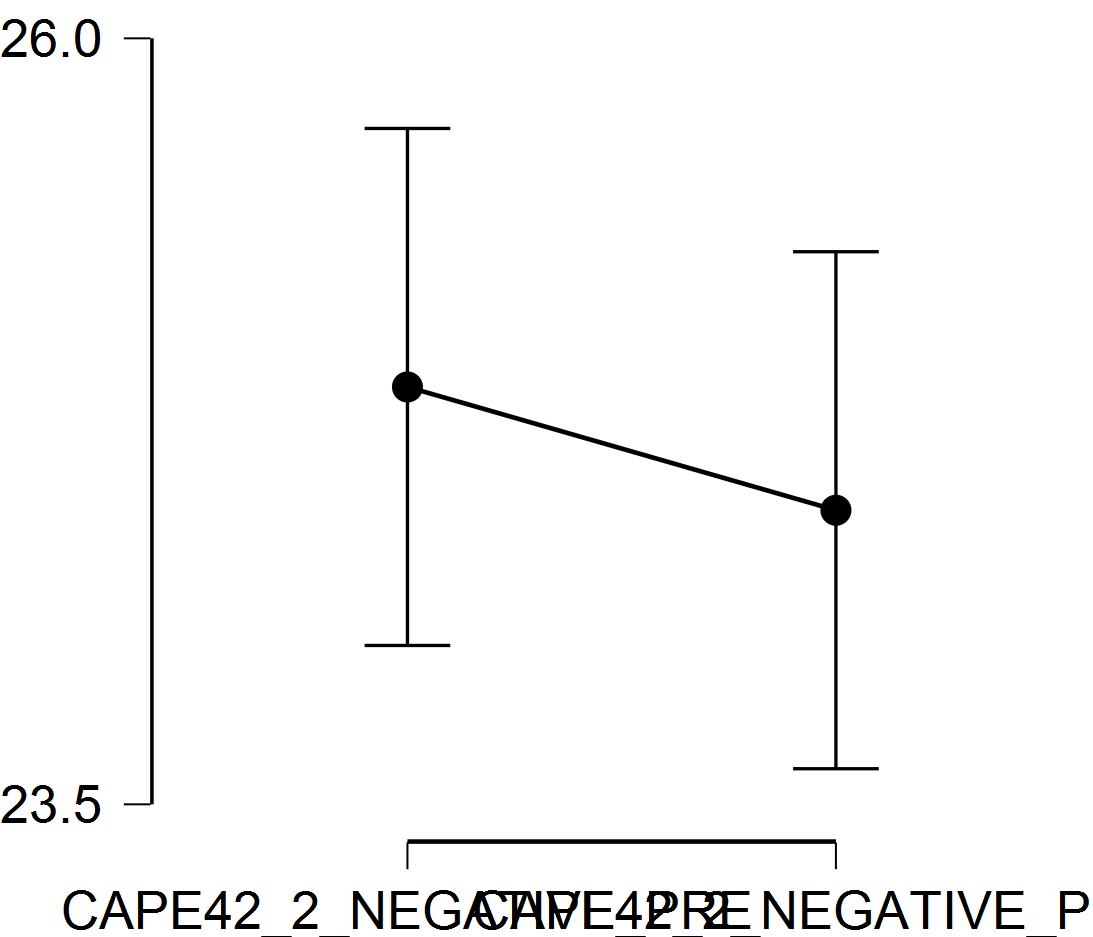

Supplement: Supplementary file 2 — Additional file 2. [file 12992_2020_603_MOESM2_ESM.jasp › resources/0/_28.png]

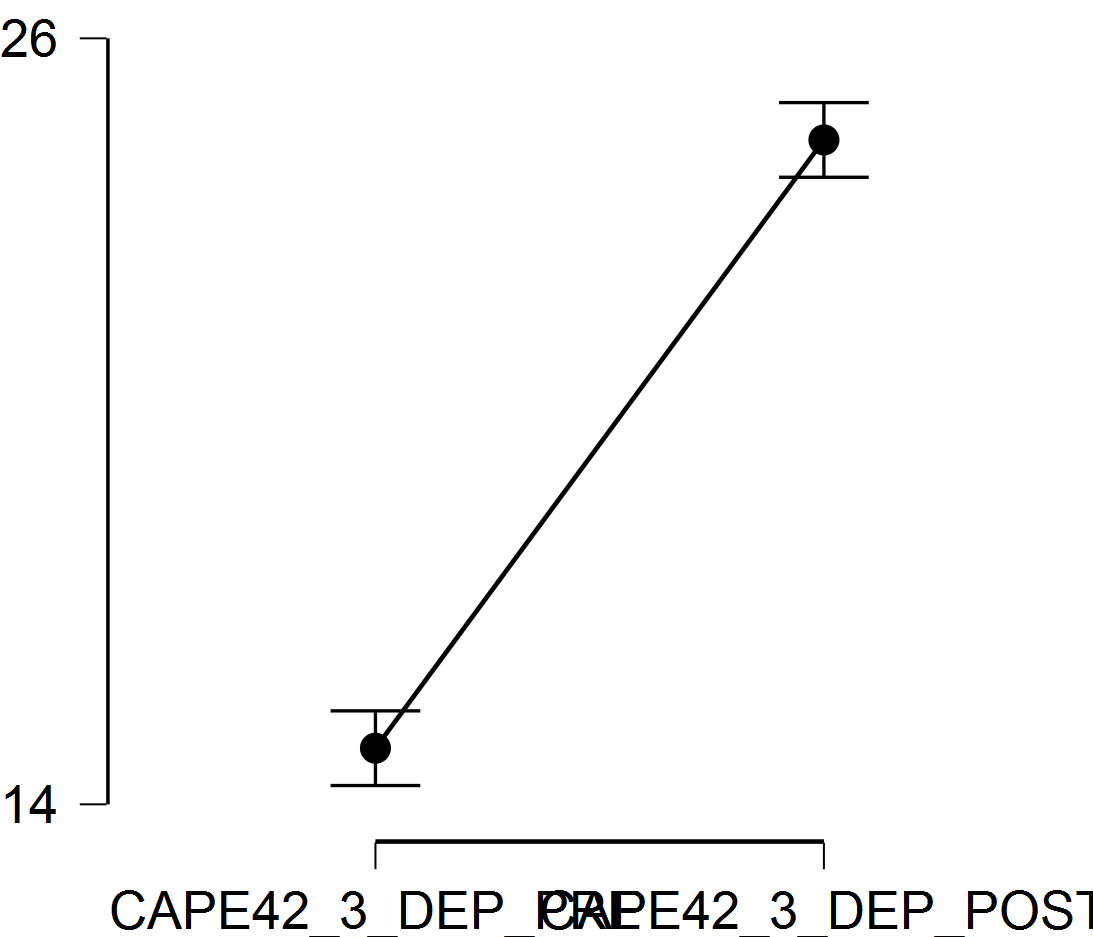

Supplement: Supplementary file 2 — Additional file 2. [file 12992_2020_603_MOESM2_ESM.jasp › resources/0/_29.png]

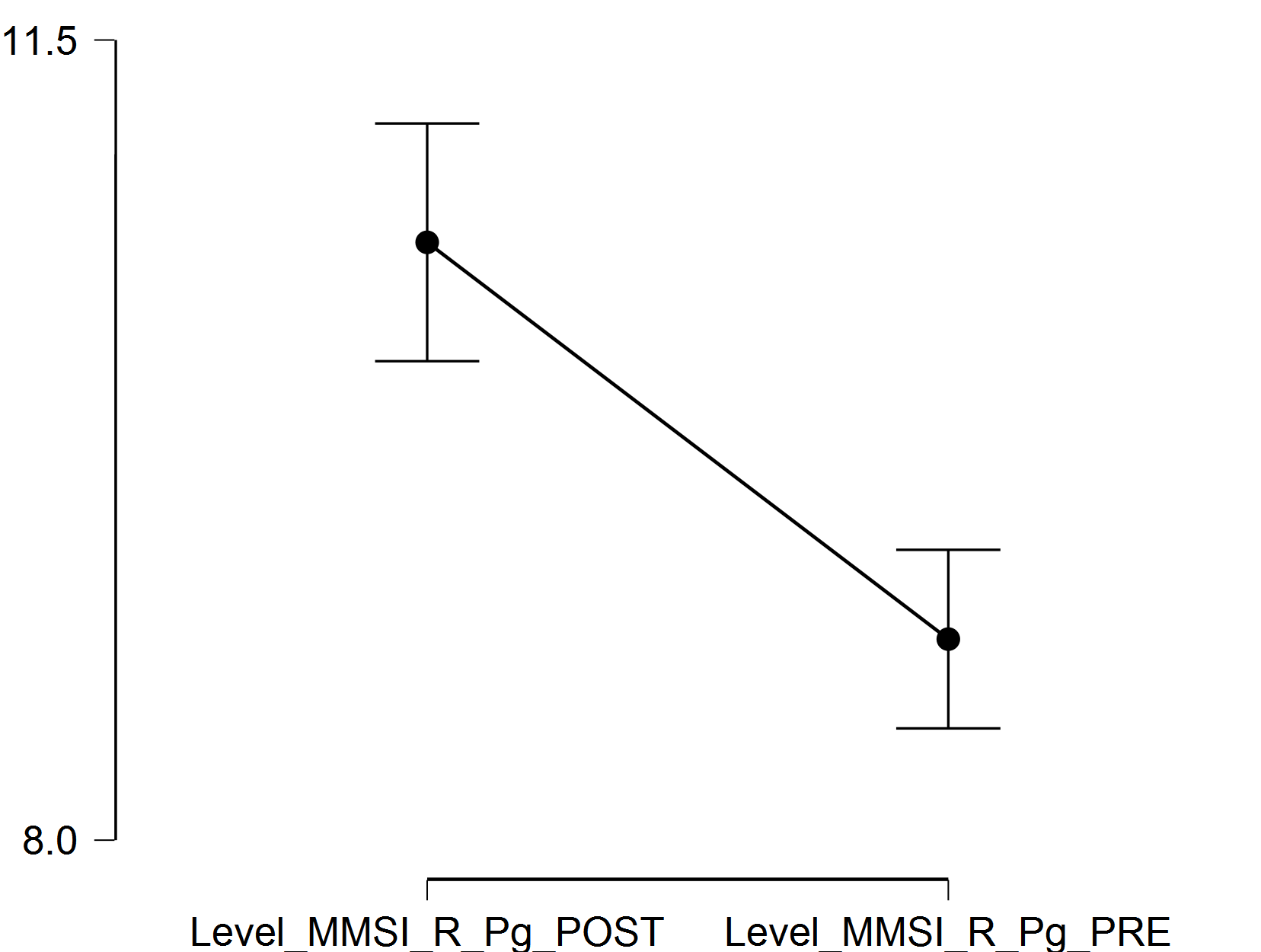

Supplement: Supplementary file 2 — Additional file 2. [file 12992_2020_603_MOESM2_ESM.jasp › resources/1/_10.png]

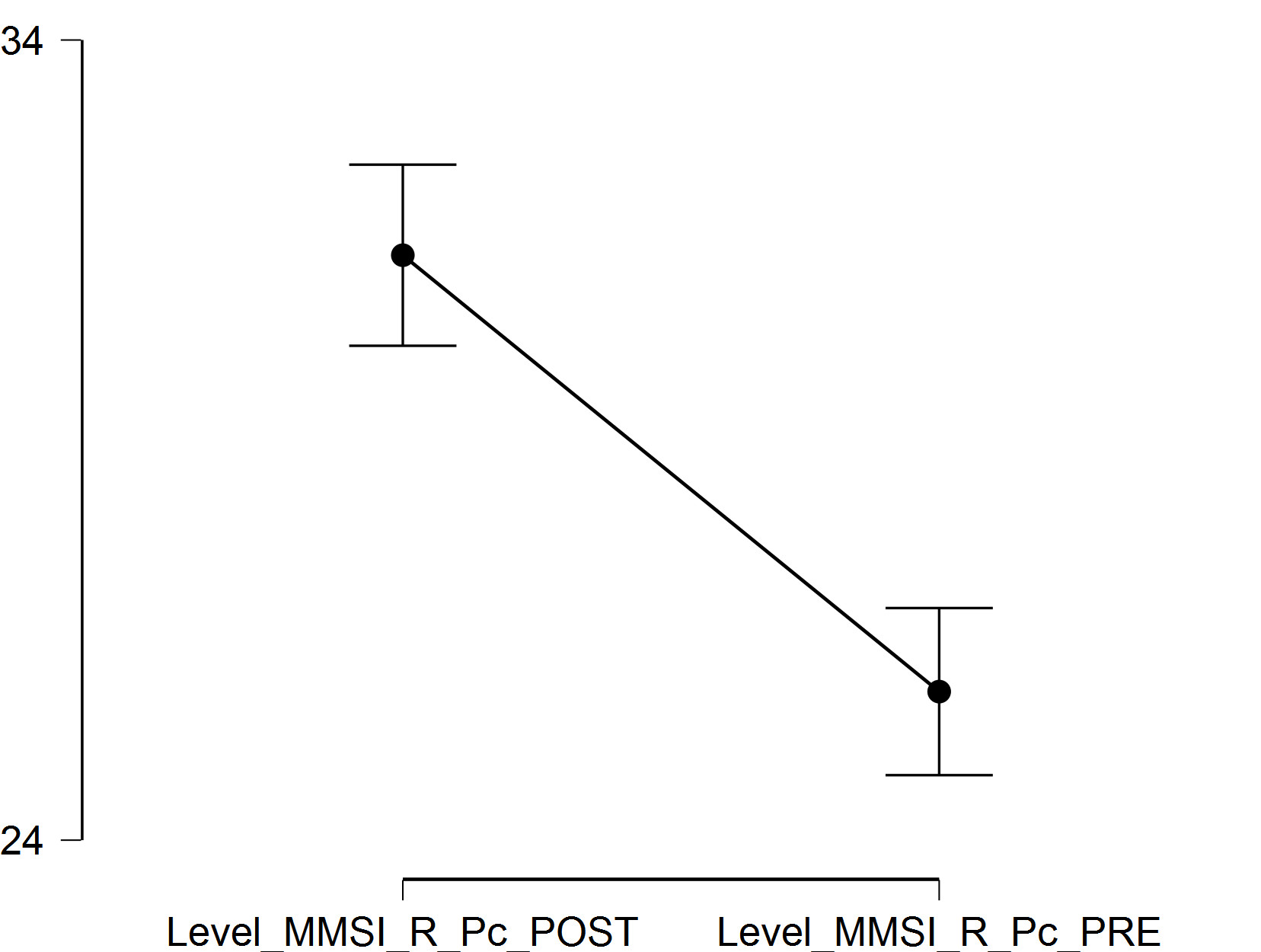

Supplement: Supplementary file 2 — Additional file 2. [file 12992_2020_603_MOESM2_ESM.jasp › resources/1/_11.png]

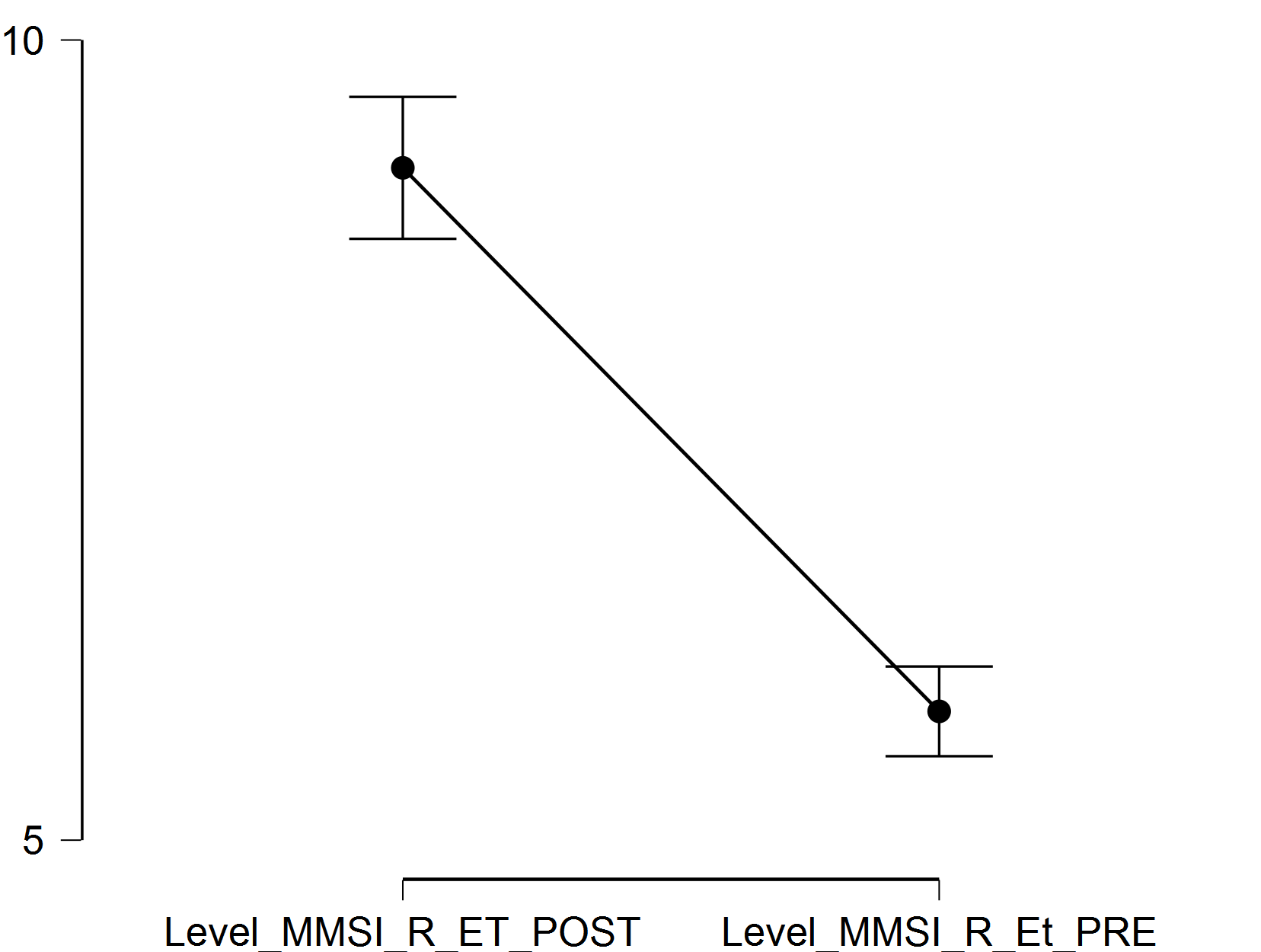

Supplement: Supplementary file 2 — Additional file 2. [file 12992_2020_603_MOESM2_ESM.jasp › resources/1/_12.png]

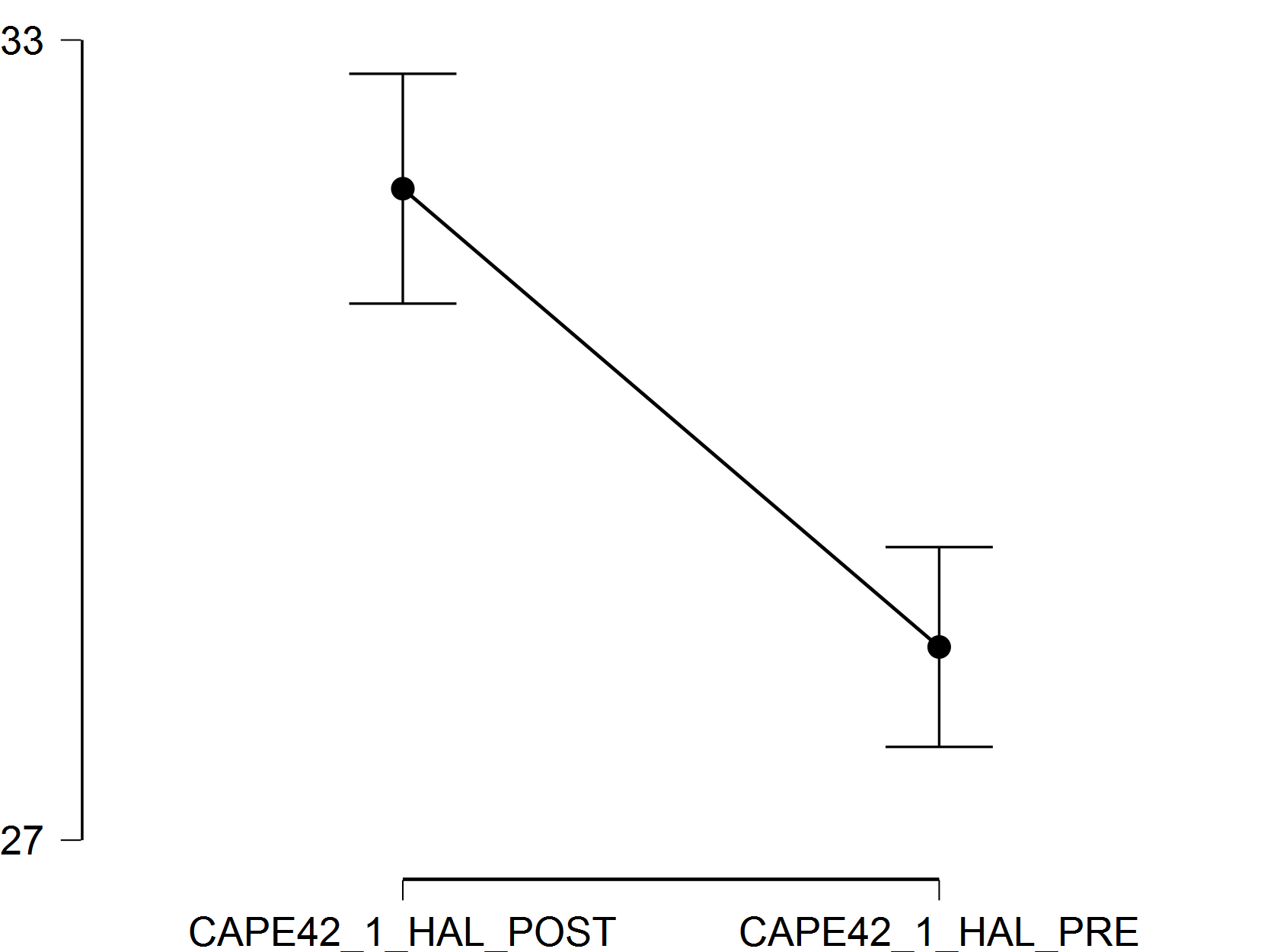

Supplement: Supplementary file 2 — Additional file 2. [file 12992_2020_603_MOESM2_ESM.jasp › resources/1/_13.png]

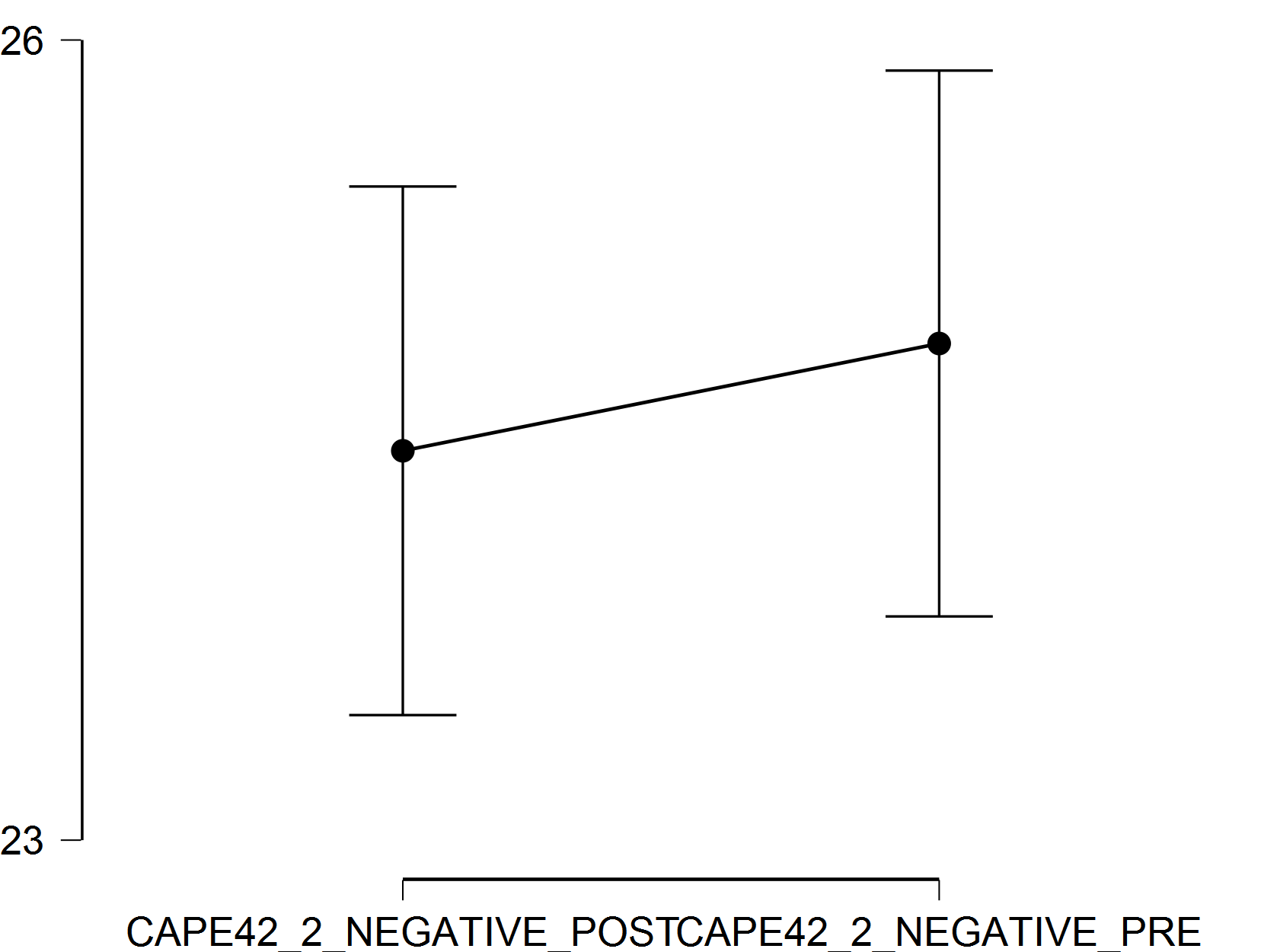

Supplement: Supplementary file 2 — Additional file 2. [file 12992_2020_603_MOESM2_ESM.jasp › resources/1/_14.png]

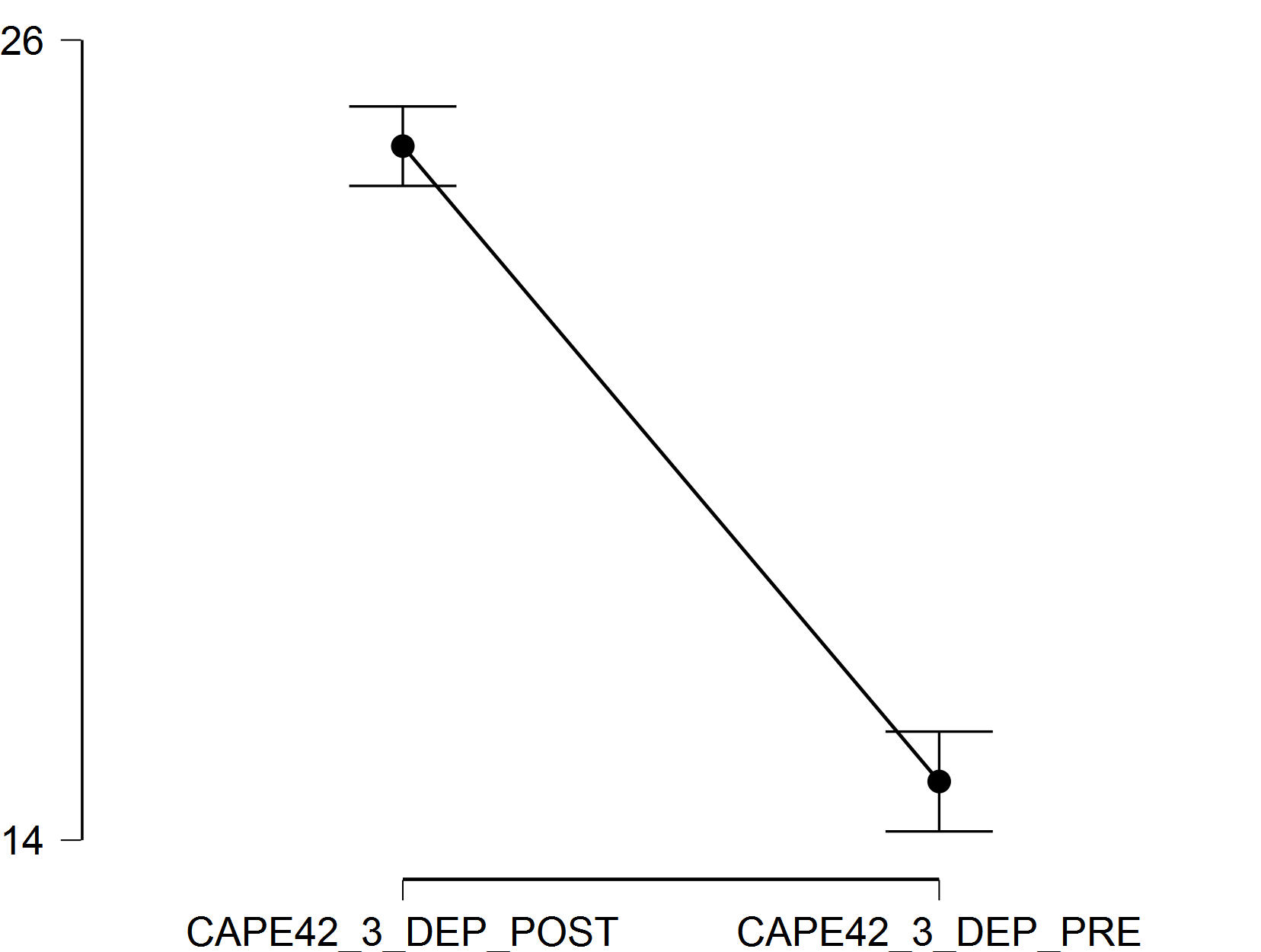

Supplement: Supplementary file 2 — Additional file 2. [file 12992_2020_603_MOESM2_ESM.jasp › resources/1/_15.png]

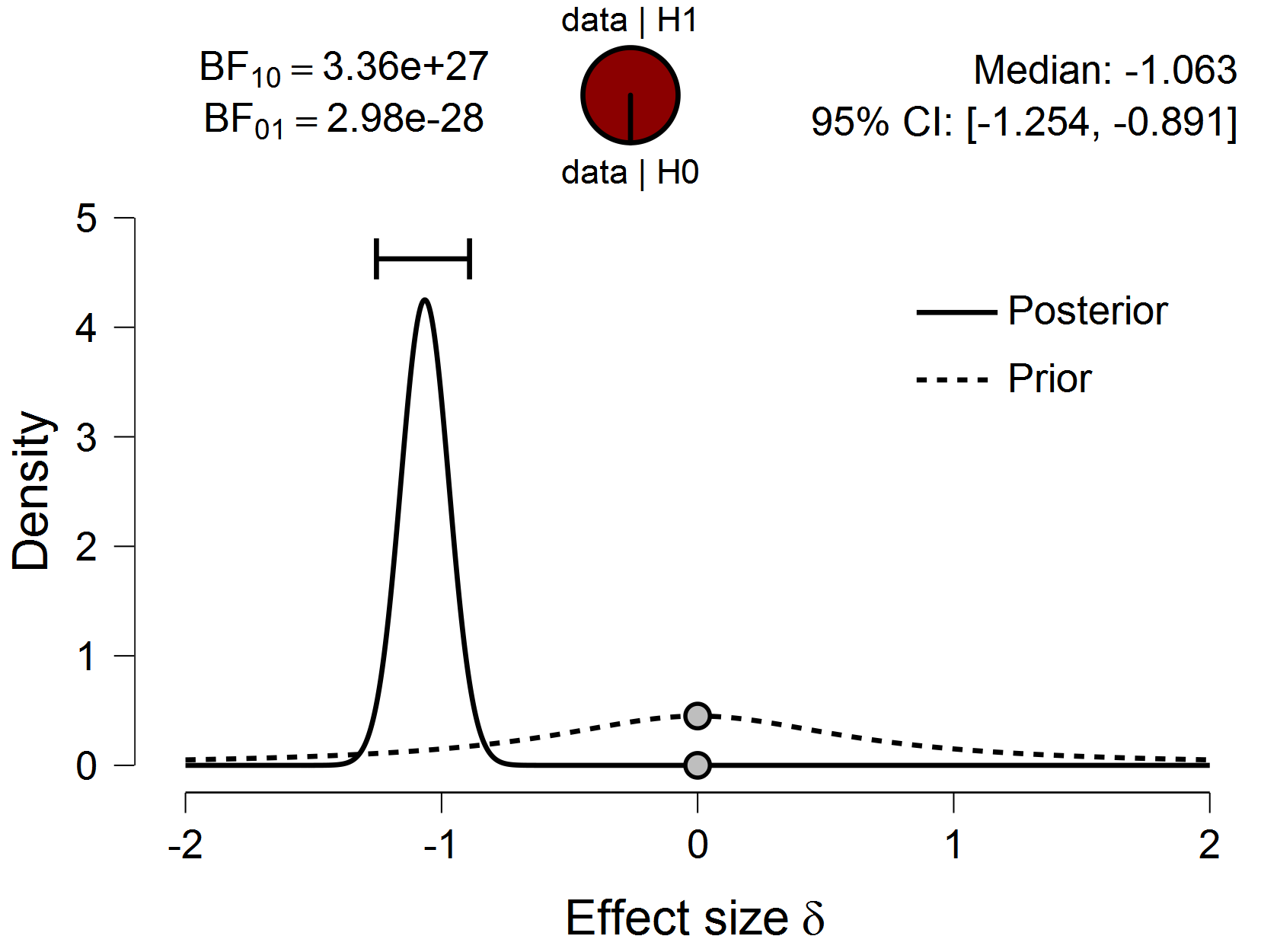

Supplement: Supplementary file 2 — Additional file 2. [file 12992_2020_603_MOESM2_ESM.jasp › resources/1/_16.png]

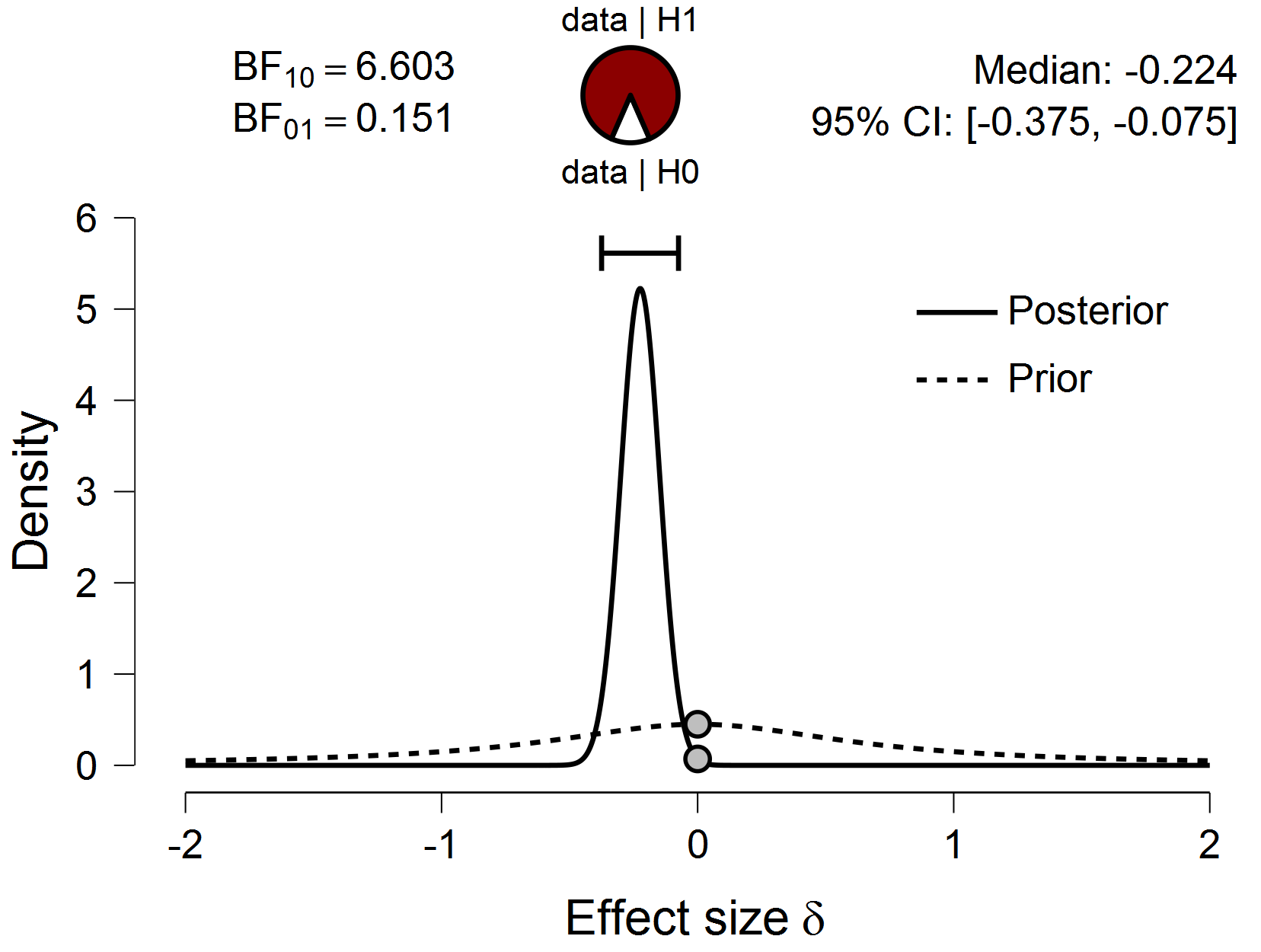

Supplement: Supplementary file 2 — Additional file 2. [file 12992_2020_603_MOESM2_ESM.jasp › resources/1/_17.png]

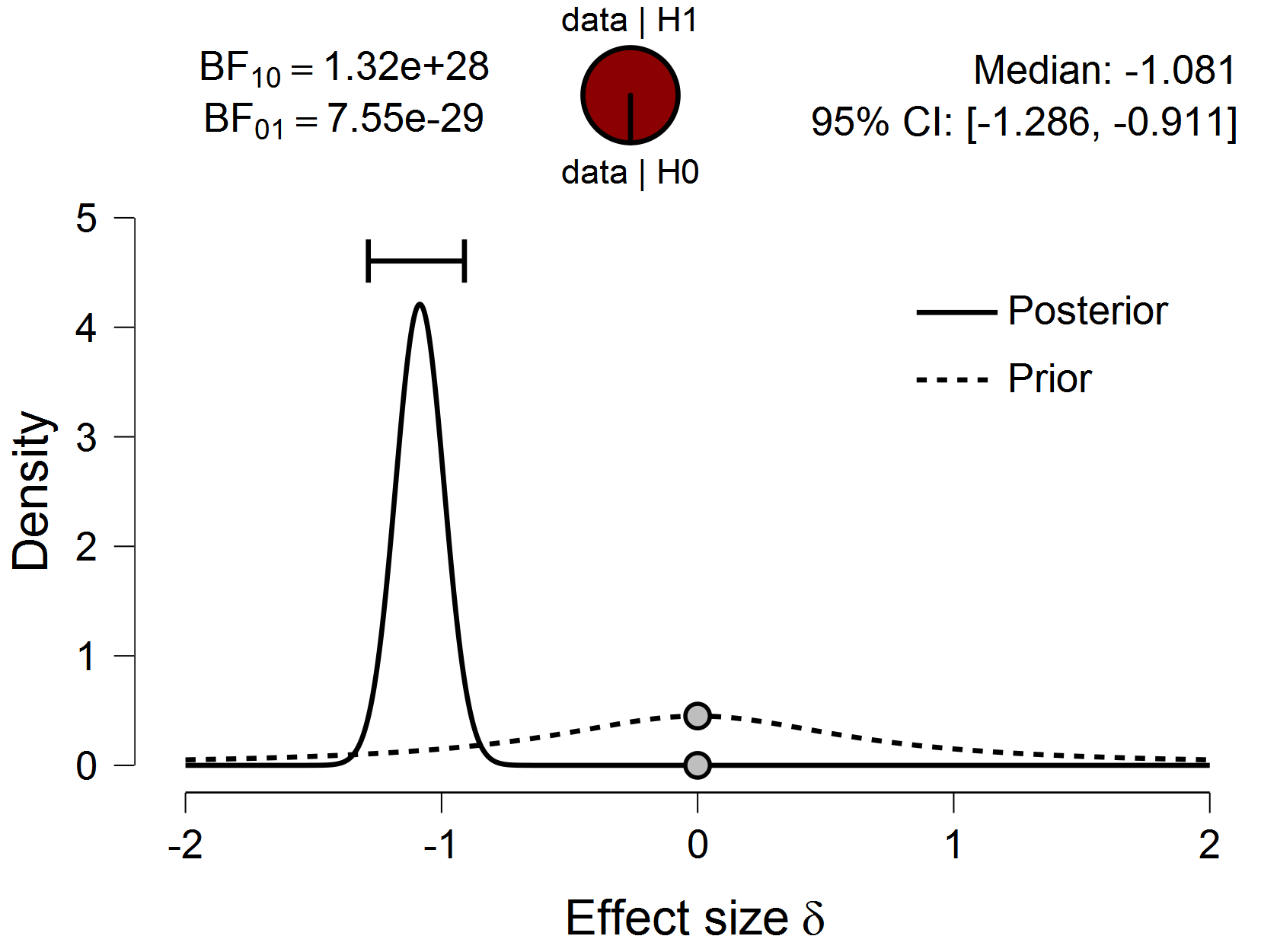

Supplement: Supplementary file 2 — Additional file 2. [file 12992_2020_603_MOESM2_ESM.jasp › resources/1/_18.png]

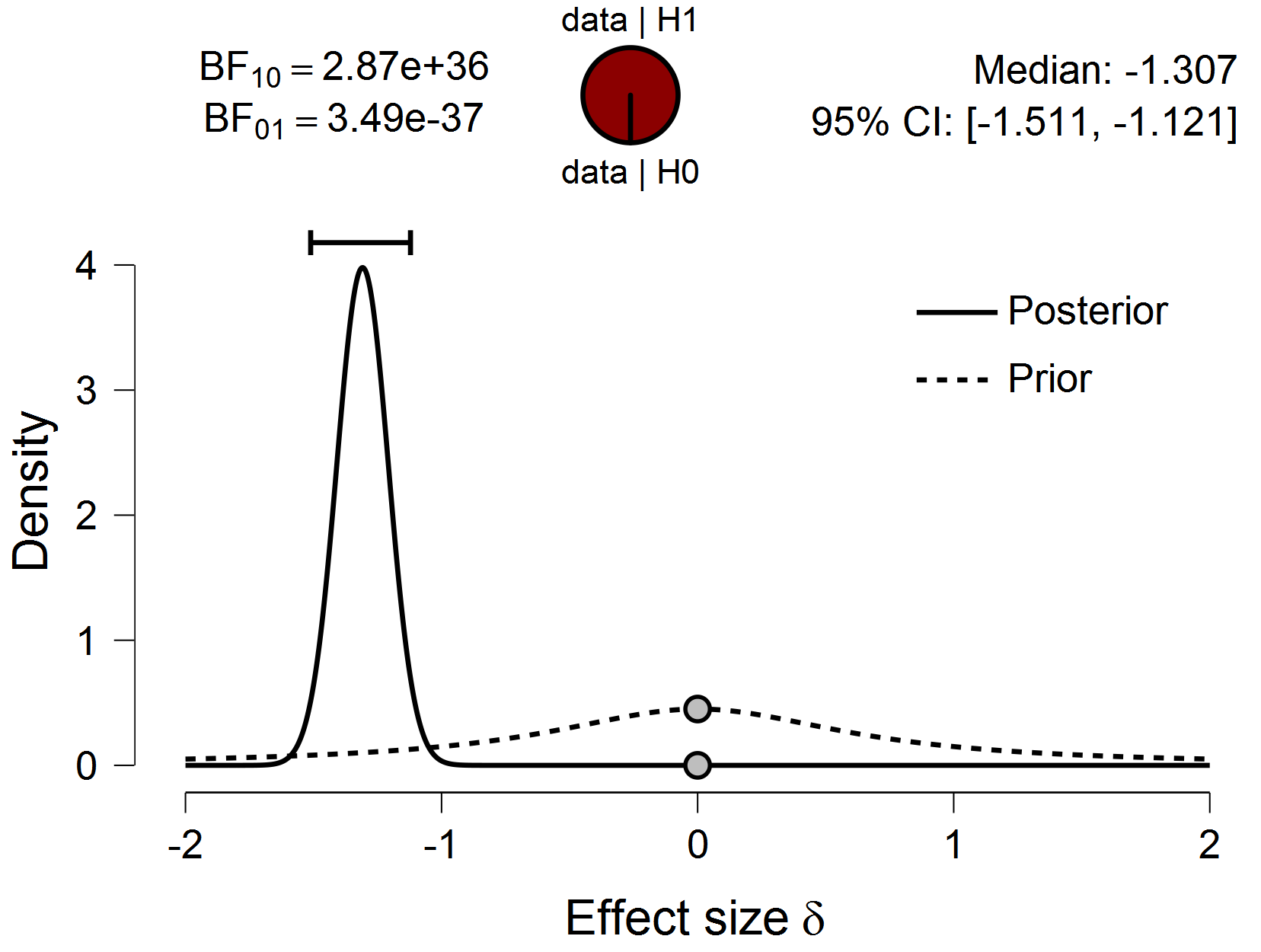

Supplement: Supplementary file 2 — Additional file 2. [file 12992_2020_603_MOESM2_ESM.jasp › resources/1/_19.png]

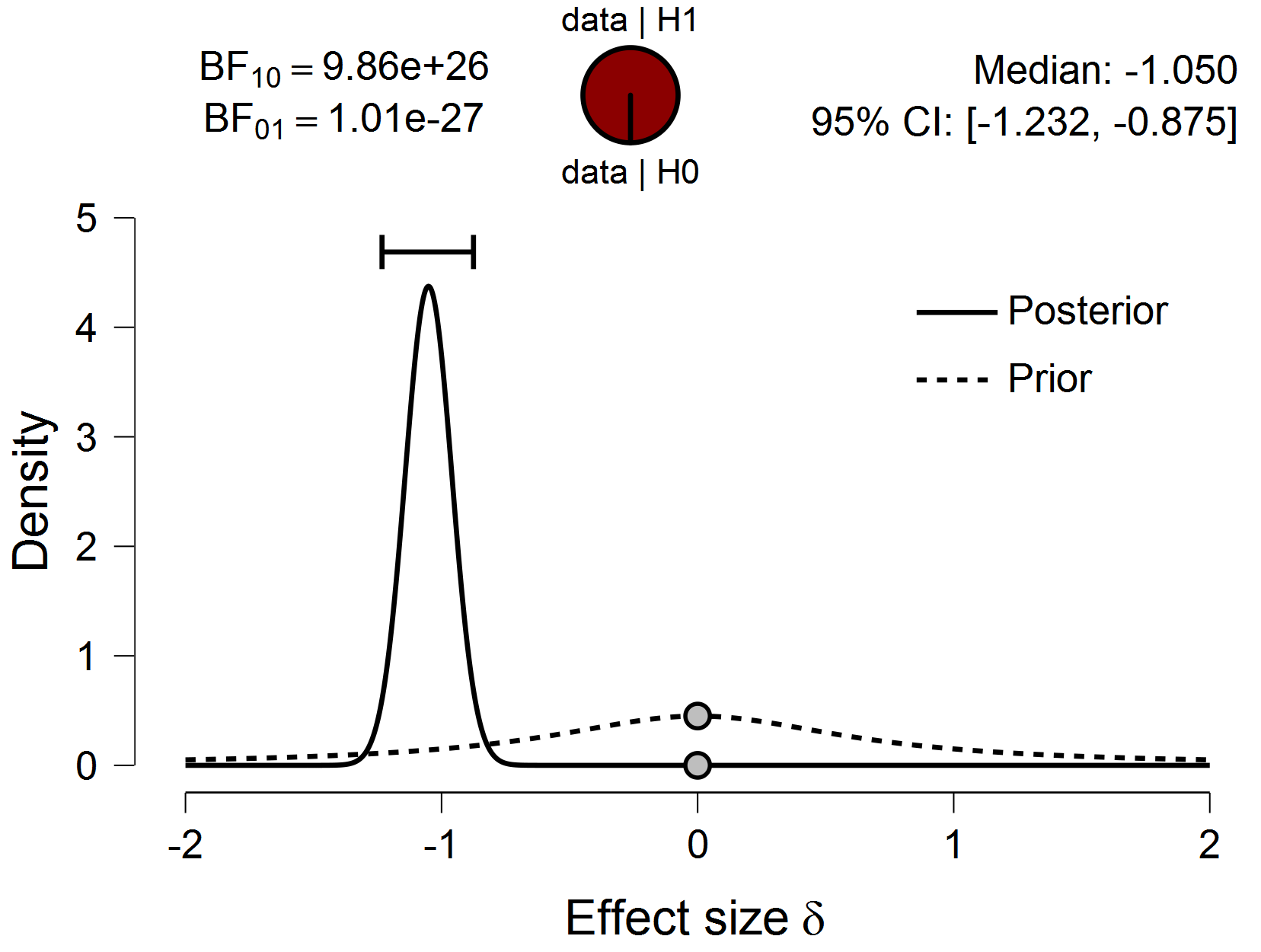

Supplement: Supplementary file 2 — Additional file 2. [file 12992_2020_603_MOESM2_ESM.jasp › resources/1/_20.png]

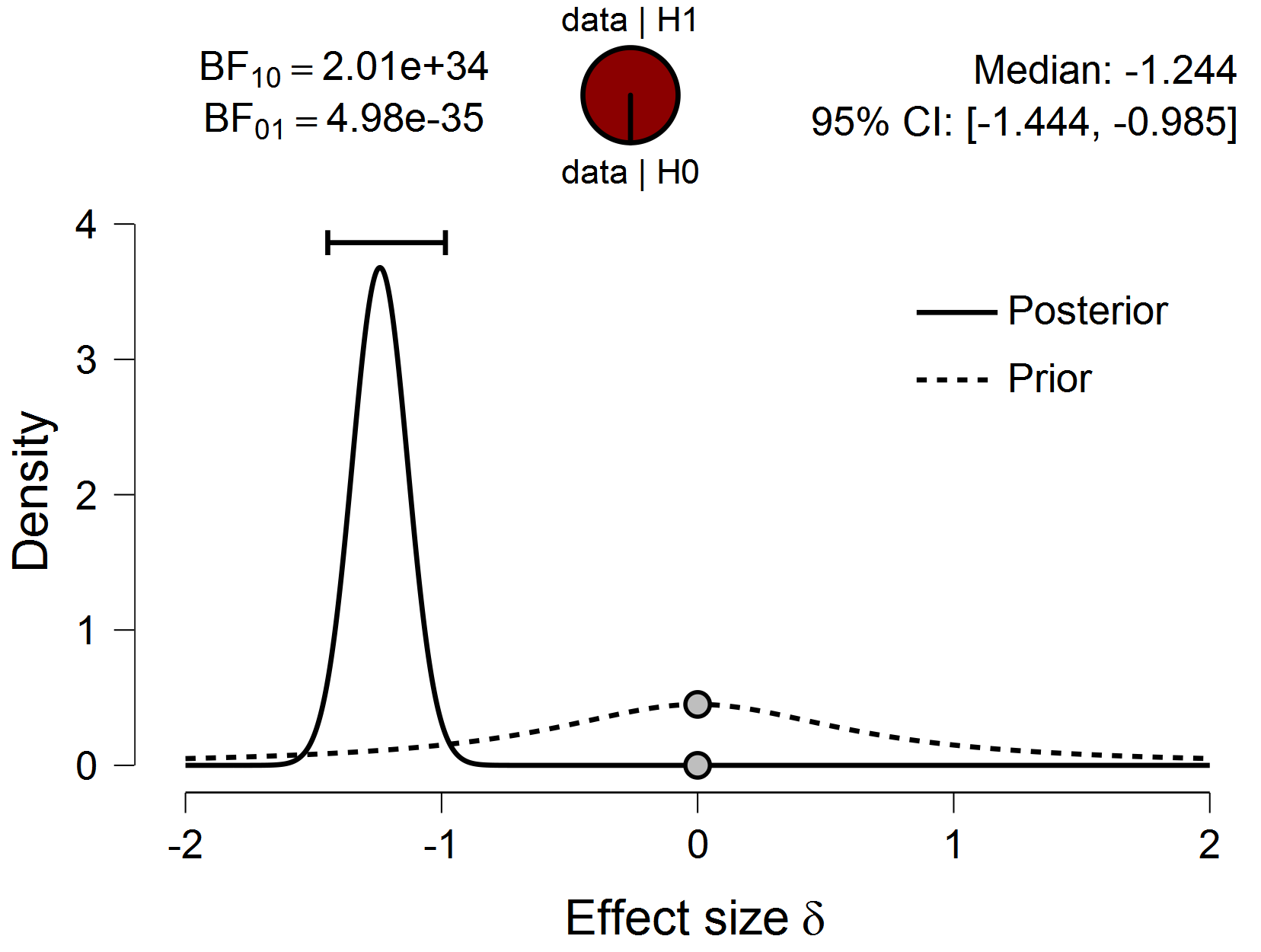

Supplement: Supplementary file 2 — Additional file 2. [file 12992_2020_603_MOESM2_ESM.jasp › resources/1/_21.png]

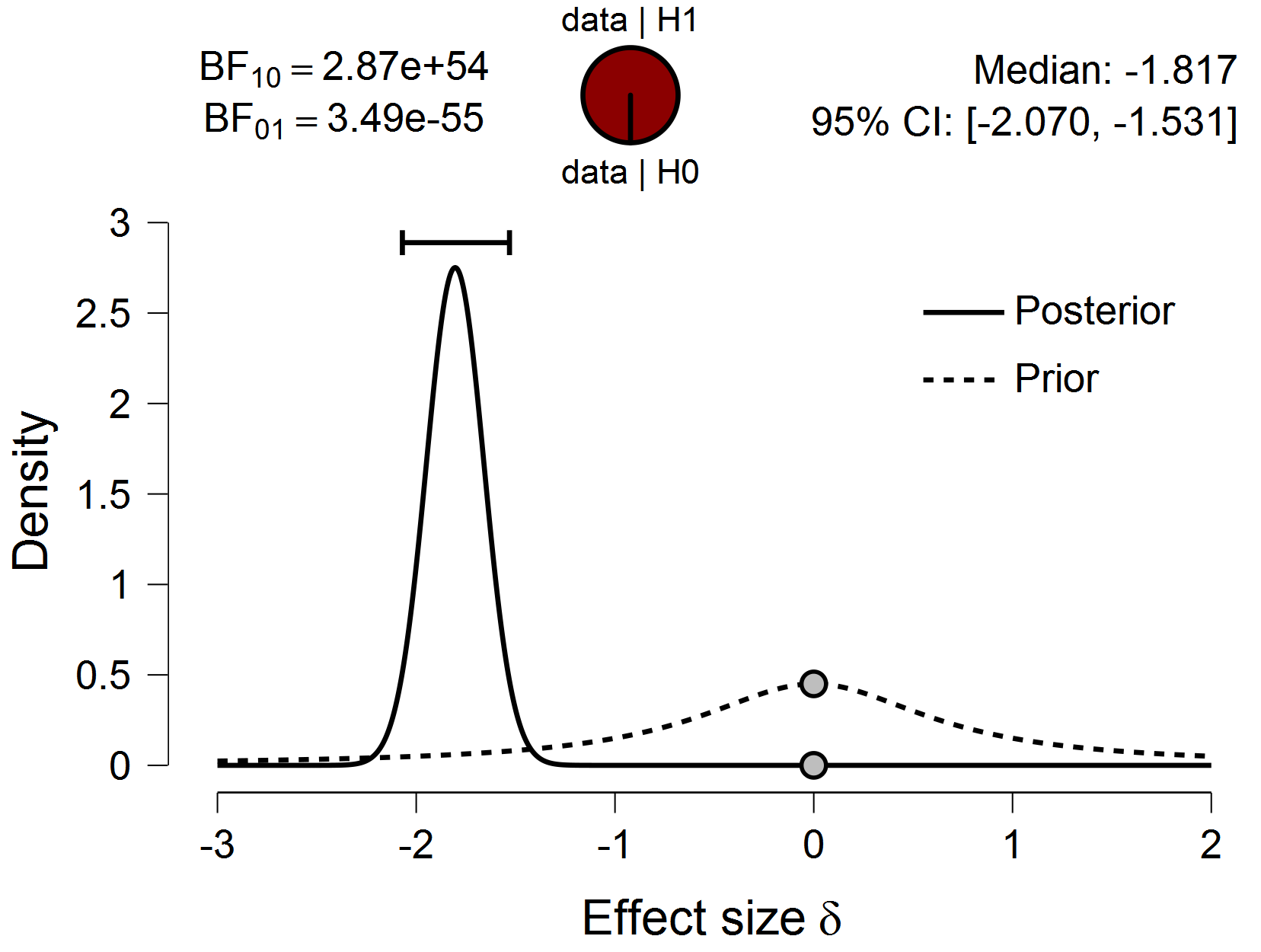

Supplement: Supplementary file 2 — Additional file 2. [file 12992_2020_603_MOESM2_ESM.jasp › resources/1/_22.png]

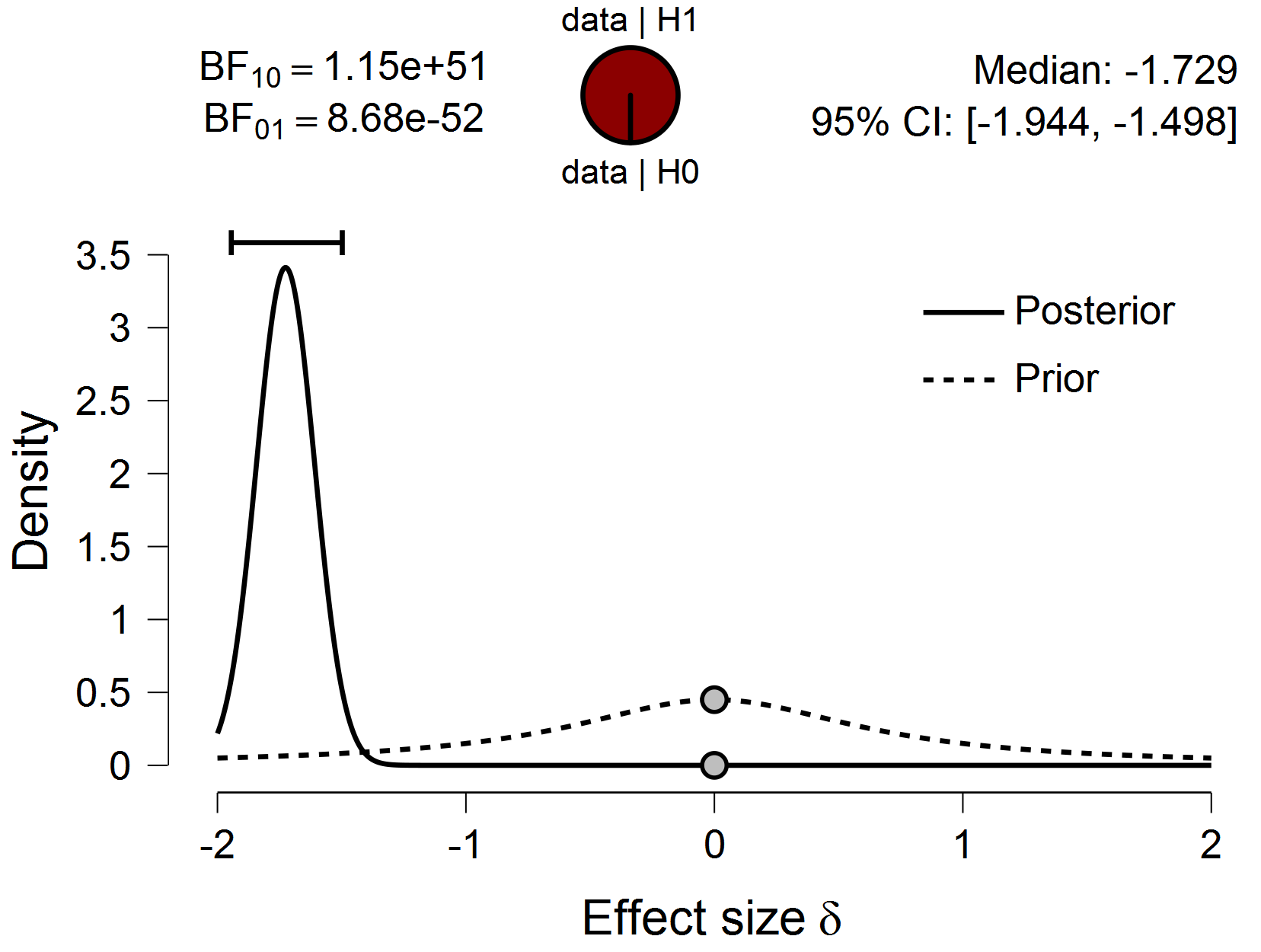

Supplement: Supplementary file 2 — Additional file 2. [file 12992_2020_603_MOESM2_ESM.jasp › resources/1/_23.png]

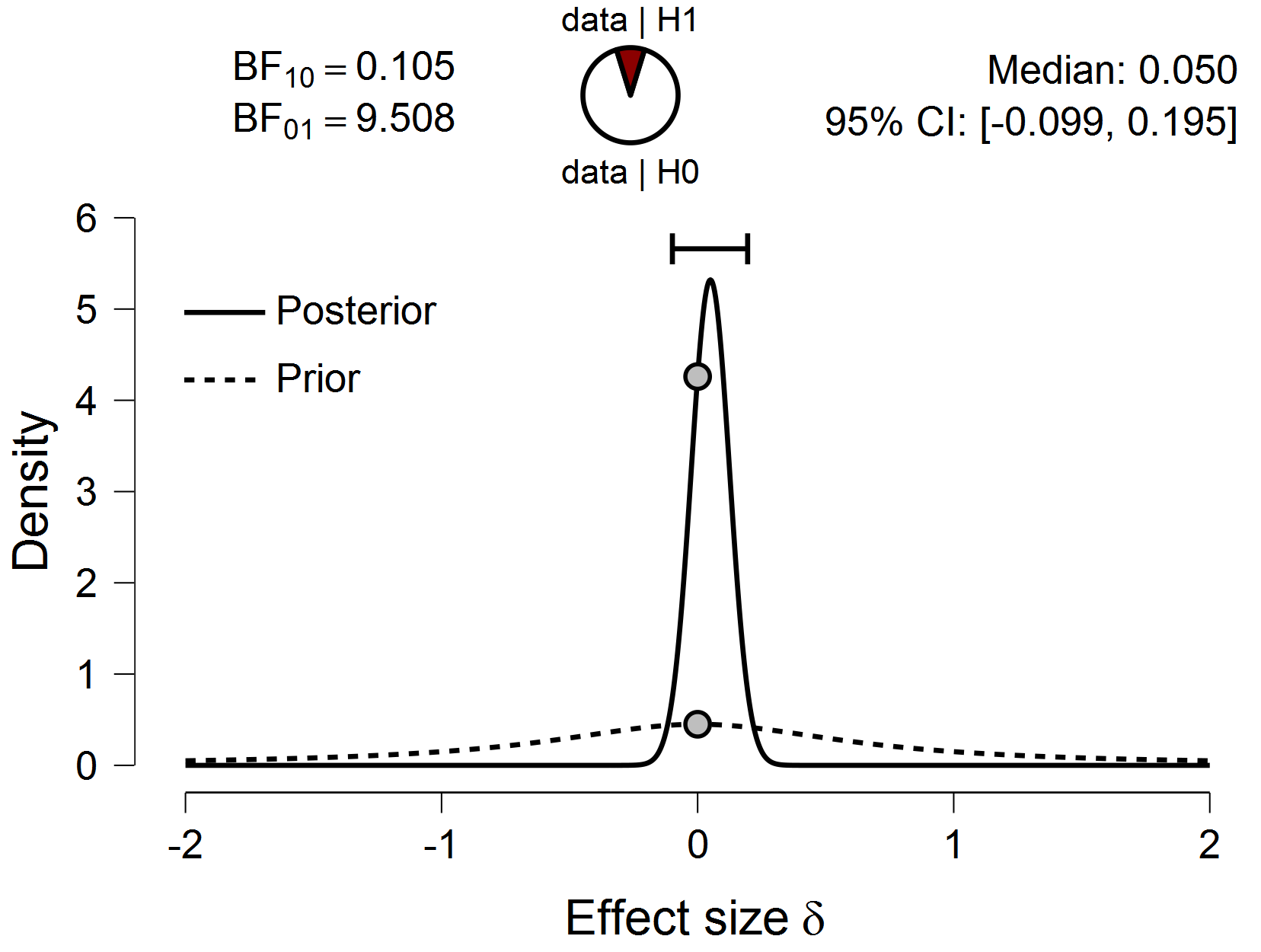

Supplement: Supplementary file 2 — Additional file 2. [file 12992_2020_603_MOESM2_ESM.jasp › resources/1/_24.png]

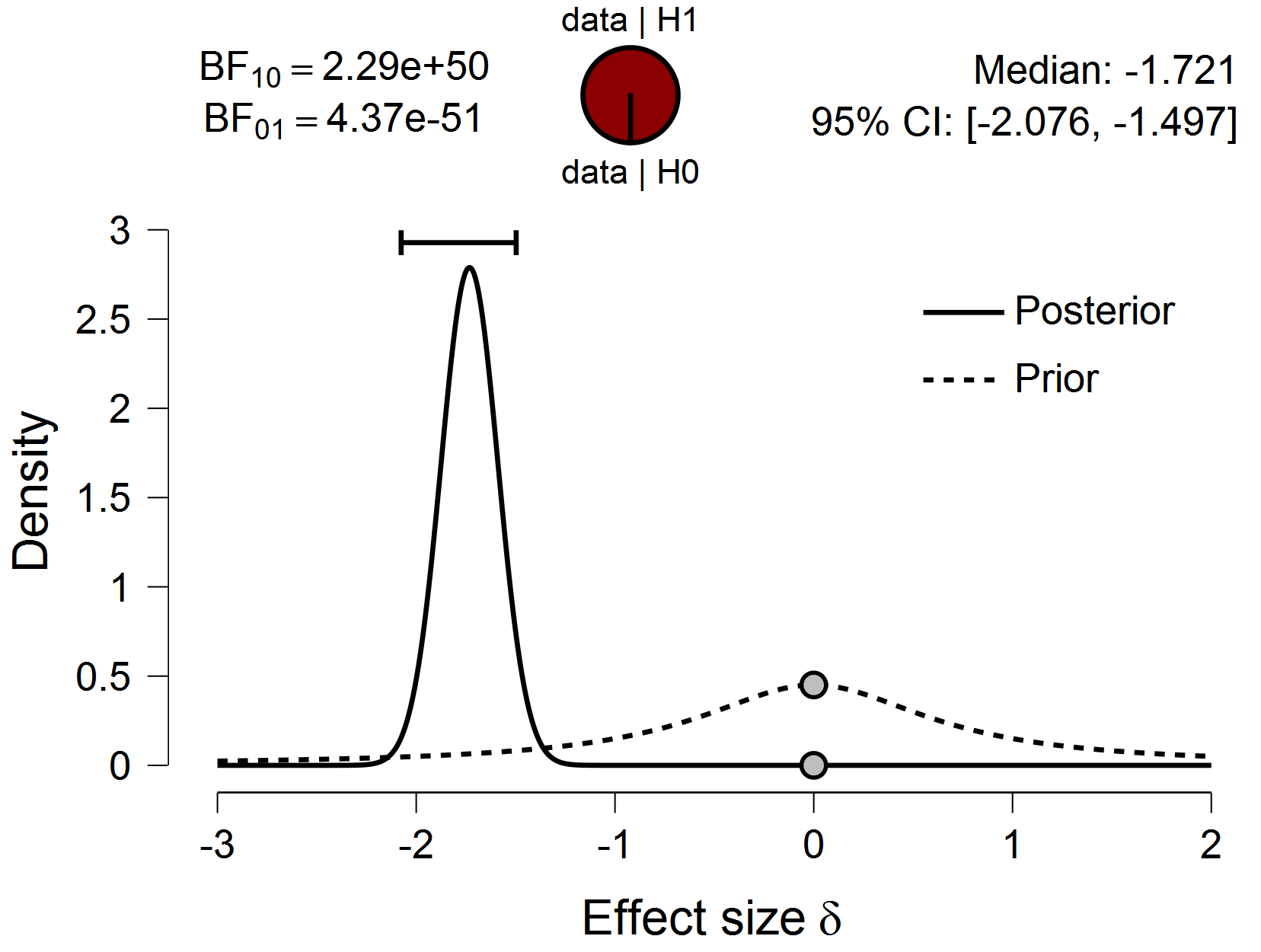

Supplement: Supplementary file 2 — Additional file 2. [file 12992_2020_603_MOESM2_ESM.jasp › resources/1/_25.png]

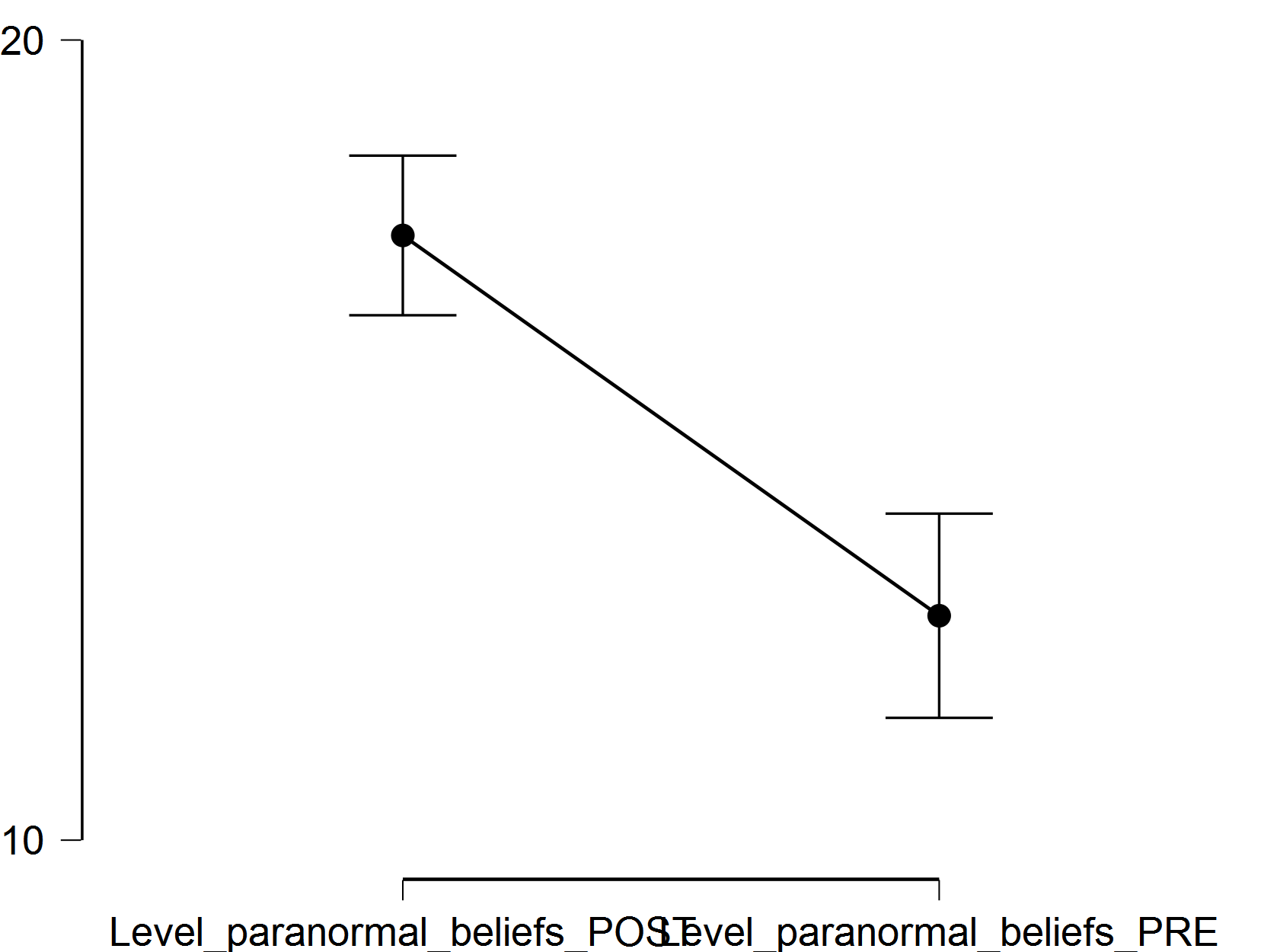

Supplement: Supplementary file 2 — Additional file 2. [file 12992_2020_603_MOESM2_ESM.jasp › resources/1/_6.png]

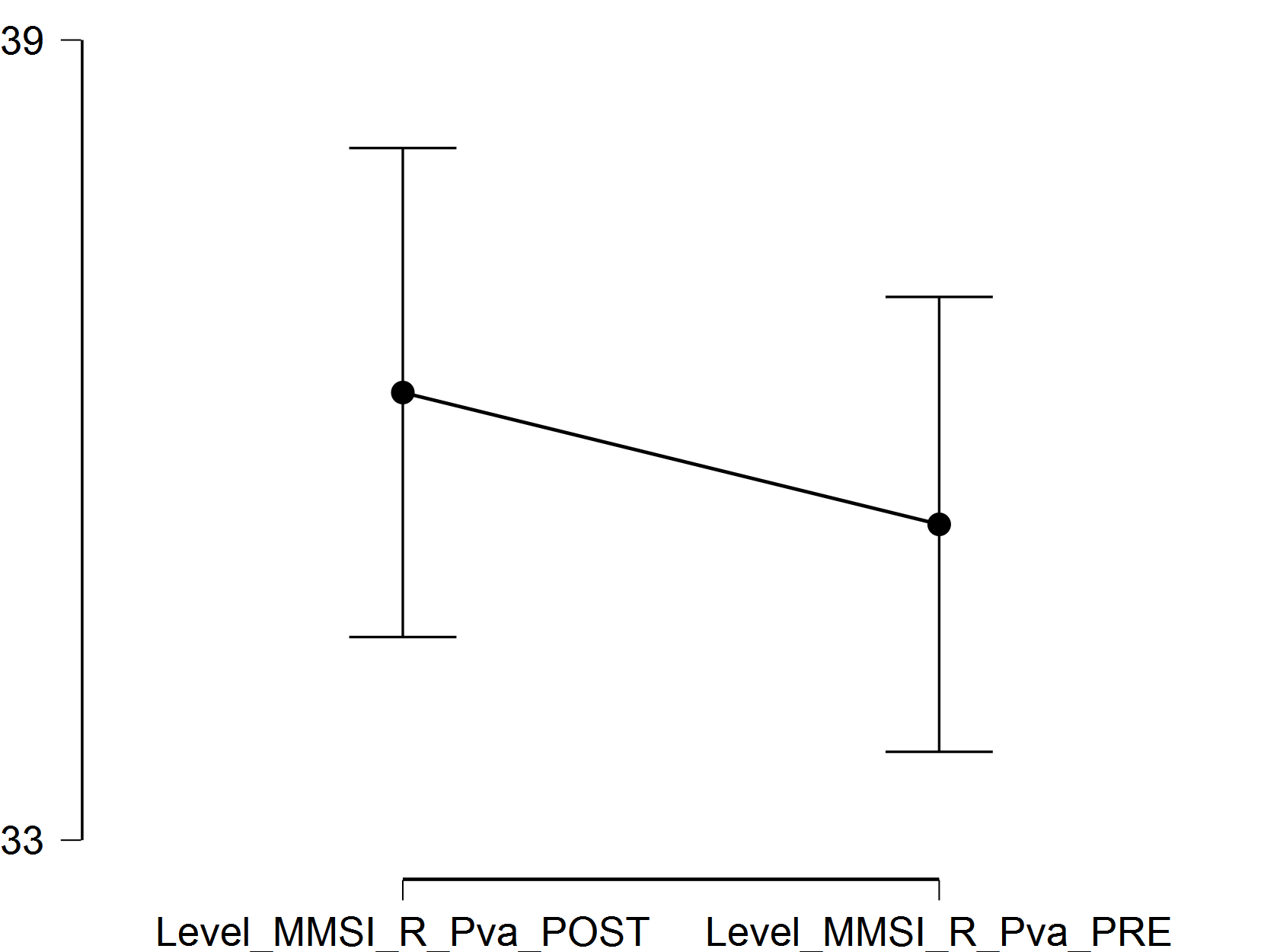

Supplement: Supplementary file 2 — Additional file 2. [file 12992_2020_603_MOESM2_ESM.jasp › resources/1/_7.png]

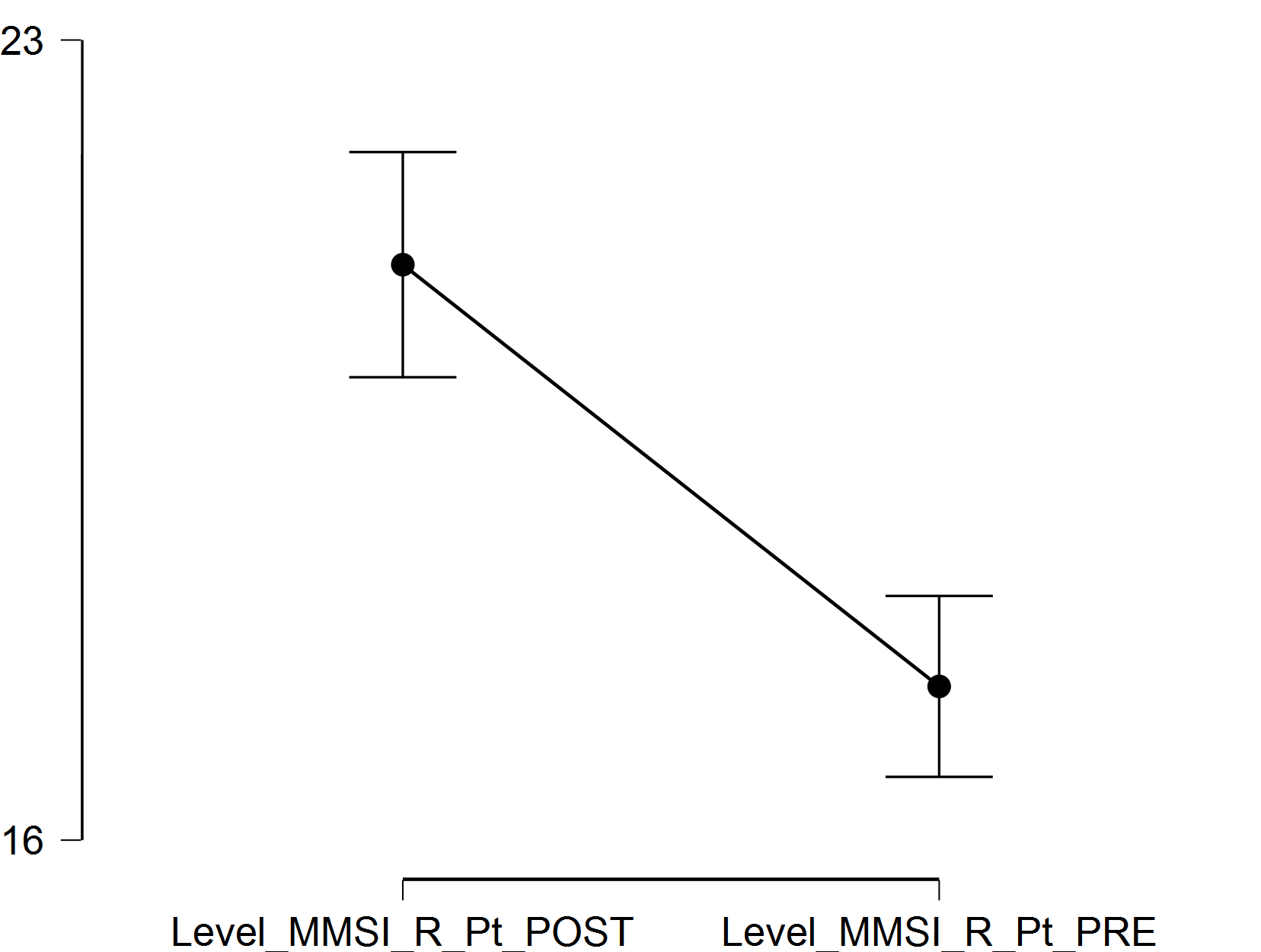

Supplement: Supplementary file 2 — Additional file 2. [file 12992_2020_603_MOESM2_ESM.jasp › resources/1/_8.png]

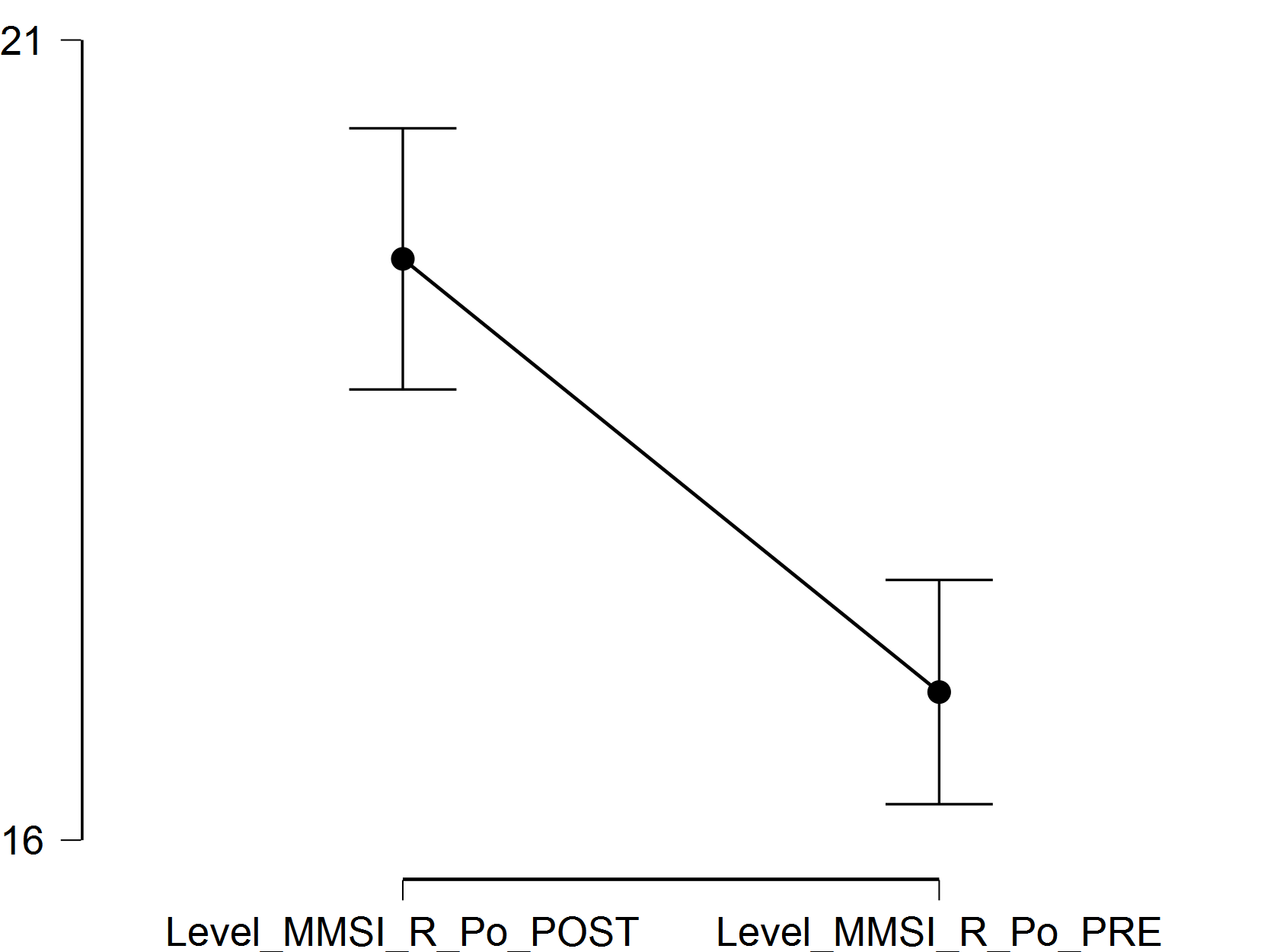

Supplement: Supplementary file 2 — Additional file 2. [file 12992_2020_603_MOESM2_ESM.jasp › resources/1/_9.png]
